# The origin and evolution of cultivated rice and genomic signatures of heterosis for yield traits in super-hybrid rice

**DOI:** 10.1186/s12915-025-02255-2

**Published:** 2025-06-04

**Authors:** Yiyong Zhao, Tao Li, Daliang Liu, Hao Yin, Liang Wang, Song Lu, Houlin Yu, Xinhao Sun, Taikui Zhang, Quanzhi Zhao

**Affiliations:** 1 Institute of Rice Industry Technology Research, College of Agriculture, Guizhou University, Guiyang, 550025 China; 2 State Key Laboratory of Public Big Data, College of Computer Science and Technology, Guizhou University, Guiyang, 550025 China; 3 Department of Biochemistry and Molecular Biology, University of Massachusetts Amherst, Amherst, MA 01003 USA; 4 College of Science, Northeastern University, Boston, MA 02115 USA; 5 Department of Biology, The Eberly College of Science, and, The Huck Institutes of the Life Sciences, The Pennsylvania State University , University Park, PA 16802 USA

**Keywords:** Rice, Phylogenomics, Gene duplications, Domestication, De novo mutations, eQTL, Heterosis

## Abstract

**Background:**

Understanding the evolutionary history of cultivated rice (*Oryza sativa*) and the genomic basis of heterosis is crucial for advancing rice productivity and ensuring global food security. The origins of the two main subspecies, *indica* and *japonica*, remain contentious, with debates over single versus multiple domestication events. Additionally, the genetic mechanisms underlying heterosis in elite super-hybrid rice varieties are not fully elucidated.

**Results:**

We performed a comprehensive genome-scale phylogenomic analysis using 33 high-quality Oryzeae genomes, integrating 39,984 gene trees. Our findings support the independent origins of *indica* and *japonica* subspecies, with molecular dating and synonymous substitution rates indicating nearly synchronous evolutionary trajectories. Analysis of 1383 gene duplications in the common ancestor of *O. sativa* revealed their involvement in vital biological processes and environmental adaptability. Phylogenomic analyses revealed no significant genomic signatures indicative of extensive hybridization events between the progenitors of *indica* and *japonica*. Newly generated 71.67 Gb of whole-genome sequencing data of five elite super-hybrid rice varieties and their progenitors uncovered differential positive selection and genetic exchanges between subspecies, contributing to heterosis formation. Transcriptome analyses highlighted the predominance of non-additive gene expression in heterosis, especially in genes related to DNA repair and recombination. Furthermore, expression quantitative trait locus (eQTL) and de novo mutation analyses identified key developmental and stress response genes, offering potential targets for enhancing heterosis.

**Conclusions:**

Our study provides robust evidence for the independent domestication of *indica* and *japonica* rice subspecies and elucidates the genomic features associated with heterosis in super-hybrid rice. By identifying key genes linked to adaptability and heterosis, we offer valuable insights and genetic resources for breeding programs aimed at improving rice yield and resilience. These findings enhance our understanding of rice evolution and the complex genetic factors driving heterosis, contributing to future strategies for agricultural productivity enhancement.

**Supplementary Information:**

The online version contains supplementary material available at 10.1186/s12915-025-02255-2.

## Background

Rice, along with maize and wheat, is one of the three principal global cereal crops, grown in diverse environments. The genus *Oryza* probably originated approximately 130 million years ago in Gondwanaland, and subsequent climatic and geographic changes led to speciation across continents, resulting in various genome types (AA, BB, CC, BBCC, CCDD, EE, FF, GG, HHJJ, and HHKK), including 20 wild species and two cultivated species, namely Asian rice (*Oryza sativa* L.) and African rice (*Oryza glaberrima*) [1–3]. Despite the generally poor agronomic traits of wild species, varieties from different growth regions exhibit distinct stress resistance and serve as reservoirs for excellent natural genetic resources [4–6].

The high yield of Asian cultivated rice has led to its widespread cultivation globally, providing about 20% of human caloric intake [7], especially in Asian countries like China, India, Japan, and Korea, where over 90% of rice cultivation and consumption occurs, providing the main source of carbohydrates and energy to billions of people [8, 9]. Previous studies have suggested that Asian cultivated rice can be primarily classified into *indica*, *japonica* (tropical *japonica* and temperate *japonica*), *aus*, and aromatic subgroups [7]. Genetic, ecogeographical, and archeological evidence highlights significant differences between the two main subspecies of Asian cultivated rice, *indica* and *japonica* [1, 10]. The differentiation between temperate and tropical *japonica* originated from adaptive evolution in distinct regions, while *aus* is more closely related to *indica*. Additionally, aromatic characteristics are closely associated with *japonica* [11].

Despite extensive research on Asian cultivated rice due to its economic importance and role as a model crop [1, 12–16], the origins of its subgroups remain unresolved; two main hypotheses persist: a single origin of *indica* and *japonica* and multiple origins. The single origin hypothesis posits that *japonica* originated in southern China and later hybridized with local wild rice in South and Southeast Asia to form *indica* [17], and they were both domesticated from *O. rufipogon*, supported by studies on key genes analyses like *prog1* and *Bh4* [18, 19] and single-nucleotide polymorphism (SNP) analyses of 630 genes [20]. In contrast, the multiple origin hypothesis suggests that Asian cultivated rice was independently domesticated from wild rice of different lineages. For example, genome-wide comparative analyses of the nuclear genomes from wild rice species *Oryza rufipogon* accessions W1943 and W0106, and the studies of chloroplast genomes derived from diverse rice populations have confirmed that *indica* and *japonica* subspecies underwent independent domestication events [21, 22]. Corroboratively, another study also proposed independent domestication of *indica*, *japonica*, and *aus* [11]. Recent studies suggest that domestication may have begun independently across regions, with beneficial alleles exchanged among groups [23]. Rice domestication remains complex and controversial, unlikely to be fully resolved by a single gene or limited methods.

Heterosis refers to the phenomenon where offspring (F_1_) from genetically distinct parents exhibit superior traits compared to both parents. This effect is widely applied in modern agriculture, significantly enhances crop yield and quality [24, 25]. The “Chinese Super-hybrid Rice Breeding Program” uses genetic diversity and heterosis to develop excellent varieties such as Liangyoupeijiu (LYP9), Y Liangyou 1 (Y1), Y Liangyou 2 (Y2), Y Liangyou 900 (Y900), and Xiang Liangyou 900 (XLY900) (Additional file 1: Table S1). At the turn of the twenty-first century, LYP9, derived from the combination of male parent Yangdao 6 (93–11) and female parent Peiai 64S (PA64S), achieved a yield potential of 9.75 ~ 10.5 t/hm^2^ [26]. The second-generation super hybrid rice Y1 achieves a yield potential of 12 t/hm^2^ by optimizing heterosis among subspecies and improving resistance [27, 28]. Y2, a third-generation super rice [29], improves the sink, source, and flow structure, with a potential of 13.9 t/hm^2^ [30]. Y900 marked a milestone in the development of fourth-generation super rice by integrating the strengths of both *indica* and *japonica*, exhibiting excellent agronomic traits that boosted its yield potential to 15.0 t/hm^2^ [31]. Following Y900, the salt-tolerant XLY900 variety, noted for its lodging resistance and high yield, reached a peak yield of 18.0 t/hm^2^ [32]. In summary, the genetic mechanisms behind the generational improvement of hybrid rice yields remain largely unknown. Ongoing success in breeding high-yielding super rice varieties offers valuable resources to study the genomic basis of yield traits.

Heterosis is underpinned by three classical hypotheses: dominance (partial and complete) [33], overdominance [34], and epistasis [35, 36]. Gene expression analysis of heterozygous hybrid F_1_ in maize supports these hypotheses [37]. Research has shown partial dominance, complete dominance, and overdominance effects across many genes, as evidenced by transcriptomic analyses. For instance, the *SFT* gene in tomato [35], *Dw1*, *Dw3*, and *qHT7.1* genes in sorghum [38], and *GS3* and *Ghd7* genes in rice [39] contribute to phenotypic superiority through dominance or overdominance effects. Advances in genomics and high-throughput sequencing, along with transcriptomic analyses, have enhanced our understanding of the molecular mechanisms behind heterosis and the relationship between allele-specific expression and hybrid dominance. Studies show that in hybrid rice, either parental allele can be advantageous under different conditions, enabling selective expression of the beneficial allele [40]. The “homo-insufficiency” model suggests that the functional insufficiency of homozygotes in certain environmental and regulatory backgrounds is key to non-additive effects (dominance and epistasis) and heterosis formation, linking hybrid dominance to genetic backgrounds [41]. These findings provide crucial insights into the genetic mechanisms behind dominance traits in hybrid cultivars.

Phenotypic polymorphisms are frequently correlated with underlying genetic variation. Genes are key intermediaries, translating genetic diversity into observable traits and contributing to phenotypic diversity, within species. Gene expression is mainly regulated by *cis*-regulatory elements near the gene of interest [42]. Trans-acting factors, such as transcription factors, and various metabolites, which may exert their regulatory influence from a remote locus, play a pivotal role in the dynamic regulation of gene expression [42]. These mechanisms regulate gene expression patterns crucial for the development, adaptation, and evolution of organisms [42]. Analysis of expression quantitative trait loci (eQTL) links quantitative traits with genetic variation, essential for understanding the genetic architecture of complex traits [42, 43]. Next-generation sequencing has led to an increase in eQTL studies. For instance, a study on human populations has identified regulatory loci for phenotypes such as hair color, shape, and thickness [44], and analysis of more than 30,000 blood samples has revealed potential drivers for more than 1000 phenotypes [45]. Studies of the early development of maize kernels identified grain-size-related genes [46]. These eQTL-related studies enhanced our understandings of the regulatory mechanisms in animals and plants, providing significant insights into how genetic variations affect gene expression and species phenotypes.

This study aims to uncover the origin of cultivated rice and the genomic signatures of heterosis for yield traits in super-hybrid rice by integrating multi-omics data. We conducted a comprehensive genome-scale phylogenomic analysis using 33 high-quality Oryzeae genomes, including two genetically distinct lineages of Asian wild rice and related genera *Leersia* and *Zizania*. This framework provided insights into the domestication process, gene flow, and hybridization dynamics, particularly among *indica* and *japonica* rice varieties. Our data suggest that the *indica* and *japonica* subspecies arose from independent domestication events, with no evidence of extensive hybridization between their progenitors. We also performed whole-genome sequencing on five leading *indica* hybrid rice varieties (LYP9, Y1, Y2, Y900, and XLY900) and their progenitors, integrating transcriptomic data for eQTL analysis to explore the genetic complexities of superior agronomic traits. Our findings indicate strong positive selection, with hybridization and introgression between super-hybrids and japonica rice. Divergent gene expression patterns revealed the significance of non-additive genes in trait dominance, and eQTL and de novo SNP analyses identified key genes linked to yield. Overall, our study offers a robust phylogenomic framework for understanding rice domestication and highlights the intricate genetic factors driving yield superiority in super-hybrid rice varieties.

## Results

### Large-scale phylogenomic profiles support the independent origins of indica and japonica subspecies

**Fig. 1:**
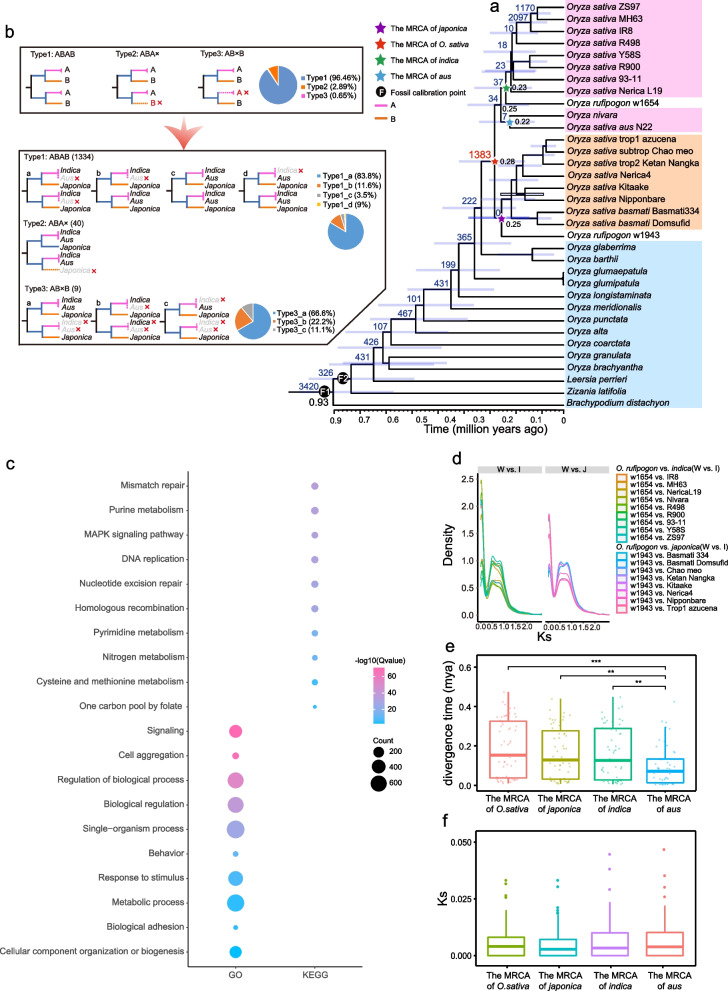
Genome-scale phylogenomic analysis and divergence among *Oryza* species. **a** Phylogenetic tree depicting the phylogenetic relationships among various rice varieties in the context of geological times. The blue bars on the node of species tree represent the 95% highest posterior density (HPD) for the estimated divergence times that all nodes in the species tree have support values greater than 0.95. The pentagrams represent the MRCA of each rice subgroup, with divergence times denoted to the right. The number of gene duplication events is indicated at the upper left of each node, with the red star marking the MRCA of *Oryza sativa*. **b** Topological classification of the duplication event in the MRCA of the *Oryza sativa* into three types, illustrated by different patterns (ABAB, ABAX, and ABXB) with corresponding pie charts showing the proportion of each type, to elucidate the evolutionary history among Asian rice subspecies. **c** This figure visualizes the top 10 ranked Gene Ontology (GO) biological processes (BP) and Kyoto Encyclopedia of Genes and Genomes (KEGG) pathways (sorted from smallest to largest *Q*-values, *Q* < 0.05) associated with genes derived from the duplications of the MRCA of *Oryza sativa*. These results underscore the functional importance of these genes in key biological processes and pathways, highlighting their critical roles in the evolutionary adaptation and development of Asian rice species. **d** Interspecific Ks density profiles of syntenic gene pairs between wild and cultivated rice species. **e** Boxplot displaying the divergence time estimates for 54 putative domestication genes from the regions of low nucleotide diversity in the MRCA of *O. sativa*, *japonica*, *indica*, and *aus* rice subgroups. **f** Boxplot showing the distribution of Ks values for 54 domestication genes across the same ancestral nodes of Asian rice subgroups as in **e**

In this study, we selected 33 high-quality genomes across various rice subgroups in Oryzeae (Additional file 1: Table S2) along with the outgroup *Brachypodium distachyon* genome for rice phylogenomic analyses at the genomic level. Our study generated an extensive set of 39,984 gene family trees, which coalesced into a highly supported species tree. Notably, members of both *indica* and *japonica* were distinctly separated into two monophyletic groups (Fig. 1a), highlighting the independent origins of *indica* and *japonica*. Our data further revealed that *O. rufipogon* w1654 and *O. rufipogon* w1943 represent the most divergent lineages within the *indica* and *japonica* subgroups, respectively. These findings are in alignment with previous studies that propose perennial wild rice *O. rufipogon* and annual wild rice *O. nivara* as the domestication ancestors of *O. sativa* [1, 17]. Intriguingly, our data also revealed that annual *O. nivara* is the sister group to the *aus* subgroup. Furthermore, the combination of *O. nivara* and *aus* emerges as the sister to the *indica* group, consistent with prior research [11]. This comprehensive phylogenomic profiling thus supports the independent origins of the *indica* and *japonica* subspecies.

The presence of Ks peaks from paralogous genes within a genome could serve as an indicator of gene duplication burst events (Additional file 2: Fig. S1b). To investigate gene duplication events, we employed Tree2GD [47] to perform phylogenomic profiling across all gene family trees, using the rooted species tree as a framework. This analysis led to the identification of a total of 1383 gene duplication events at the MRCA node of *O. sativa*, as depicted in Fig. 1a. The gene tree topologies for these 1383 gene duplications were categorized into ABAB, ABA, and ABB types (Fig. 1b), accounting for 96.46%, 2.89%, and 0.65% of the total, respectively. The dominant prevalence of gene duplications retained in the ABAB type suggested the absence of a hybridization signal among the MRCAs of the *indica*, *japonica*, and *aus* subspecies groups.

To further clarify the mechanism underlying the 1383 gene duplication (GD) events observed at the ancestral node of Asian cultivated rice (Fig. 1a), we conducted comprehensive syntenic dot plot and Ks analyses for Nipponbare (representing the *japonica* subgroup) and 93–11 (representing the *indica* subgroup). The absence of a syntenic block with a smaller Ks value negates the possibility of a recent whole genome duplication event in the MRCA of *Oryza sativa* (Additional file 3: Fig. S2c-d). In the genomes of the representative species Nipponbare and 93–11, the retained gene duplication events from the aforementioned 1383 GDs involved 402 and 81 gene duplicated pairs, respectively, which exhibit weak collinearity (Additional file 1: Table S3). Specifically, Nipponbare and 93–11 account for 0.25 and 1.23% of the gene duplication events with syntenic evidence, respectively (Additional file 3 Fig. S2a-b). Furthermore, the proportions of tandem gene duplication events in Nipponbare and 93–11 account for 3.23 and 20.98%, respectively (Additional file 3: Fig. S2e-f and Additional file 1: Table S3).

In summary, tandem duplication significantly contributes to the occurrence of gene duplication events in the MRCA of *Oryza sativa*. Additionally, a total of 359 and 218 unique genes under gene duplication events were identified in the MRCA of *Oryza sativa* in Nipponbare and 93–11, respectively (Additional file 1: Table S4). A chi-square test for genes with transposable elements (TEs) within a 2-kb region upstream and downstream of the coordinates revealed a significant correlation (*P*-value < 0.05) between the duplicated genes and TE distribution. This suggests that TE insertions may also contribute to the origin of the gene duplication burst at the ancestral node of cultivated Asian rice. Collectively, these findings support the notion that these GDs did not arise as a result of allopolyploidy. This is consistent with the pattern that the majority of GDs in the cultivated rice ancestor were ABAB topology type, suggesting that the cultivated rice ancestor likely did not undergo a hybridization event among different subspecies ancestors. However, it is plausible that there might have been a minimal number of gene introgression or gene flow events.

### Large-scale gene duplicates retained from the MRCA of Oryza sativa may contribute to environmental adaptation

Gene duplication events are critical in providing raw material for the evolution of gene novelty and complexity. In the MRCA of *Oryza sativa*, a comprehensive reconciliation of gene family trees with the species tree revealed a total of 1383 gene duplication events (refer to Fig. 1a). The analyses used GO and KEGG databases to explore the functional implications of 24,916 genes derived from 1383 gene duplications in the MRCA of *Oryza sativa*, with annotation across 20 *Oryza sativa* and their progenitors *Oryza rufipogon* genomes by EggNOG-mapper [48].

Our results revealed a total of 15 significant enriched GO terms, comprising two molecular functions (MF), two cellular component (CC), and 11 biological processes (BP) terms (Fig. 1c, Additional file 1: Table S5). Notably, biological processes such as the signaling (GO:0023052) and cell aggregation (GO:0098743) were significantly enriched. Building upon these findings, the KEGG pathway enrichment analysis provided further insights into the functional relevance of these gene duplications. A total of 27 significantly enriched KEGG pathways were identified, highlighting critical roles in both metabolic and signaling pathways (Fig. 1c, Additional file 1: Table S6). Notably, pathways such as mismatch repair (ko03430), purine metabolism (ko00230), and MAPK signaling pathway (ko04011) were among the most enriched, suggesting their importance in maintaining genomic stability, nucleotide biosynthesis, and signal transduction, respectively. These results underscore the adaptive significance of gene duplications in the MRCA of *Oryza sativa*, as they contribute not only to essential biological processes but also to species-specific traits. Furthermore, the enrichment of pathways related to DNA repair and replication implies a potential link between gene duplication events and mechanisms safeguarding genomic integrity. This comprehensive analysis provides a foundation for further exploration of the evolutionary and functional implications of these duplication events.

### Molecular clock and Ks analysis reveal parallel evolution of Indica and Japonica domestication

Our molecular dating indicated that the divergence times among ancestral wild rice lineages were ordered as: *Oryza rufipogon* w1943 > *Oryza rufipogon* w1654 > *Oryza nivara*. An analysis of the Ks density distribution of orthologous gene pairs between species in each subpopulation and their recent common ancestor revealed that Ks in the *indica* and *japonica* combinations exhibited similar distribution trends and peaks (Ks values ranging from 0.5 to 1) (Fig. 1d). In addition, we displayed boxplots of the Ks values of the MRCA of the *japonica* and *indica* subspecies in combination with the subspecies representative varieties Nipponbare vs *O. rufipogon* w1943 and 93–11 vs *O. rufipogon* w1654, respectively (Additional file 2: Fig. S1a). This clearly showed that the Ks values of the *indica* and *japonica* groups were similar, while the *aus* group was the smallest, suggesting that the *aus* subspecies formed much later than *indica* and *japonica*, whose subspecies established simultaneously. Compared to natural selection, domestication expedites the stabilization of specific traits in certain populations, thereby reducing nucleotide diversity in the corresponding sequence regions [49]. Among the previously reported nucleotide low-diversity regions located in the *japonica*, *indica*, and *aus* subgroups [11], we screened 54 putative domestication-associated genes across all species (see Methods). To assess the relative timing of domestication and to mitigate potential systematic errors introduced by concatenating multiple gene sequences, and we conducted molecular clock analyses on the concatenating of 54 OGs. This was done by concatenating all genes as well as analyzing each gene individually. Subsequently, we performed boxplot statistics on the domestication times of individual genes. The results indicated that there was no significant difference in the domestication time between *indica* and *japonica* rice, and the *aus* values were relatively small (Fig. 1e, Additional file 2: Fig. S1c and Additional file 1: Table S7). Meanwhile, the results of the Ks distribution of 54 putative domestication-associated genes in different subgroups showed that the Ks values were similar in the *indica* and *japonica* groups, but the *aus* group did not show a consistent trend out of the molecular clock (Fig. 1f, Additional file 1: Table S8), In general, the onset of domestication was similar in the MRCA of *indica* and *japonica* subspecies.

### Ancient introgression from early divergent species of Oryzeae may contribute to rice speciation

To further investigate the possibility of hybridization events between the ancestral nodes of *indica* and *japonica*, we utilized HyDe [50] to analyze hybridization and introgression signals. For HyDe analysis, we both considered orthologous genes and genes derived from gene duplication at the MRCA of *Oryza sativa*. We established two gene sets for hybridization analysis. The first set is a supermatrix derived from the concatenation of 669 orthologous groups (OGs) with a coverage rate of 50% for *Oryza sativa* samples in the species tree of this study and all these single-copy orthologous genes were split and pruned from the 1383 gene duplication (GD) events that retained in the MRCA of *Oryza sativa*. The second dataset comprises a supermatrix generated by concatenating 1300 orthologous groups (OGs) with 100% taxa coverage, representing orthologues across species within our phylogeny (Fig. 1a, Additional file 5 Fig. S4). The two datasets previously mentioned were employed in hybridization analysis to clarify the ancestral state of *Oryza sativa* and assess the extent of genetic introgression. Our study of orthologous genes (OGs) retained from duplicated genes at the MRCA of rice revealed no hybridization signals for the MRCA of *indica*, *japonica*, and *aus* subpopulations (Additional file 5: Fig. S4 a, c, e and g). However, introgression analysis involving 1300 orthologs demonstrated introgression signals from the *japonica* subpopulation to *indica* (Additional file 5: Fig. S4b, d, f, and h), indicating that *japonica* genetics were incorporated into *indica* subgroups post the divergence of their common ancestors (Additional file 5: Fig. S4 d). This finding aligns with the practices of modern hybrid breeding, where genes from *japonica* are introduced into indica through hybridization, illustrating a historical precedent for current breeding strategies.

In addition, in our study, the ancestors of the two major subgroups of cultivated rice contained genetic signals from ancient wild rice of the genus *Oryza* (Additional file 5: Fig. S4 b, c, d and f), suggesting that that introgression from ancient divergent species of Oryzeae may have played an important role in facilitating the speciation of cultivated rice. Meanwhile, these wild rice species include BB (*Oryza punctata*) and CCDD (*Oryza alta*) types of wild rice genomes, a result which broadens previous suggestions that genetic introgression between AA types of wild rice exist in African wild rice, genetic introgression between only *Oryza glumaepatula* and *Oryza barthii* in African wild rice [51].

**Fig. 2:**
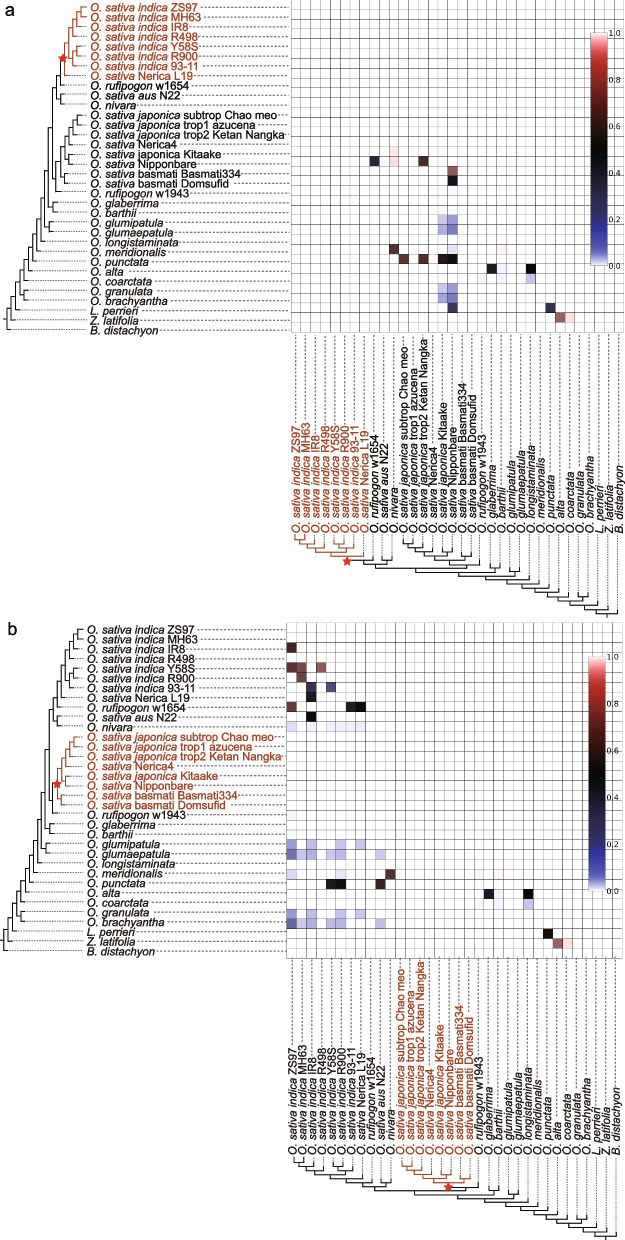
Detection of hybridization and introgression signals using HyDe in Asian cultivated rice subpopulations. **a** The heatmap depicts hybridization signals within the *indica* lineage based on 1300 single-copy orthologous genes. **b** The heatmap illustrates hybridization signals within the *japonica* lineage, also based on 1300 single-copy orthologous genes. Each cladogram highlights the focal clade with a red star. Small blue squares within the heatmap indicate interspecific gene flow signals identified by HyDe, with color intensity reflecting inheritance probabilities from taxa listed along the left axis. Clusters of small squares with identical color intensity that form larger squares, whose corresponding taxa are monophyletic, suggest that their most recent common ancestor could represent one of the parental lineages. Adjacent to each heatmap is a schematic representation of the inferred phylogenetic network derived from the cladogram and heatmap data, with numerical values denoting inheritance probabilities

**Fig. 3:**
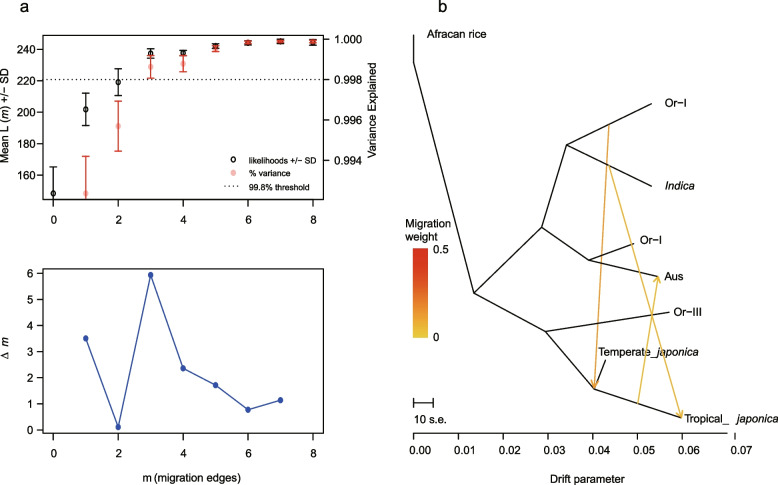
Migration dynamics and gene flow networks in Asian cultivated rice subpopulations. **a** The graph illustrates the optimal migration influx ascertained using the OptM package, with Δm indicating the identification of three significant migration events. **b** A phylogenetic network inferred by TreeMix outlines the pattern of migrations between subpopulations. The arrows, annotated in colors proportional to the ancestry contributions, direct from the contribution (donor) to receiving (recipient) subpopulation, thus mapping the route of gene flow

Furthermore, to explore the relationship between *indica* and *japonica* rice within Asian cultivated rice, we examined the introgression relationship between the *indica* and *japonica* subspecies in the analysis of 1300 single-copy OGs. As in previous studies [22, 23], we found a large amount of contributing genetic material between the *japonica* and *indica* subpopulations (Fig. 2a, b) suggesting that genetic introgression between these two subspecies is very frequent through model hybrid rice breeding procedures. On the other hand, we analyzed some *japonica*, *indica*, and wild rice genomic next-generation sequencing (NGS) data collected from previous studies (Additional file 1: Table S9) to obtain 35,502 high-quality SNPs and conducted a genetic network analysis of these species’s SNPs using Treemix [17, 52], and simulations were run with 0–8 migration events (each migration event was repeated five times). Based on the previous described method, the best number of migrations (m) was estimated among populations in the dataset [53], with results show that the variance explained and likelihood values stabilize at ∆m = 3 (Fig. 3a), suggesting that three migrations are the best fit. Specifically, observations included migration signals from the ancestral *Oryza rufipogon* to *japonica*, gene flow from *japonica* to *aus*, and migration from *indica* to *japonica*, indicating shared genetic material among subtypes (Fig. 3b). Considering the relatively minimal inferred weights associated with these three migration signals, it is posited that such patterns can be attributed to genetic introgression among *Oryza sativa* populations, as opposed to hybridization events. This interpretation underscores the subtle yet significant gene flow that characterizes the genetic landscape of these populations, distinguishing introgressive events from the more substantial genomic mergers typically associated with hybridization.

### Analysis of genomic mutation sites unveils differential positive selection across five indica super-hybrid rice varieties

**Fig. 4:**
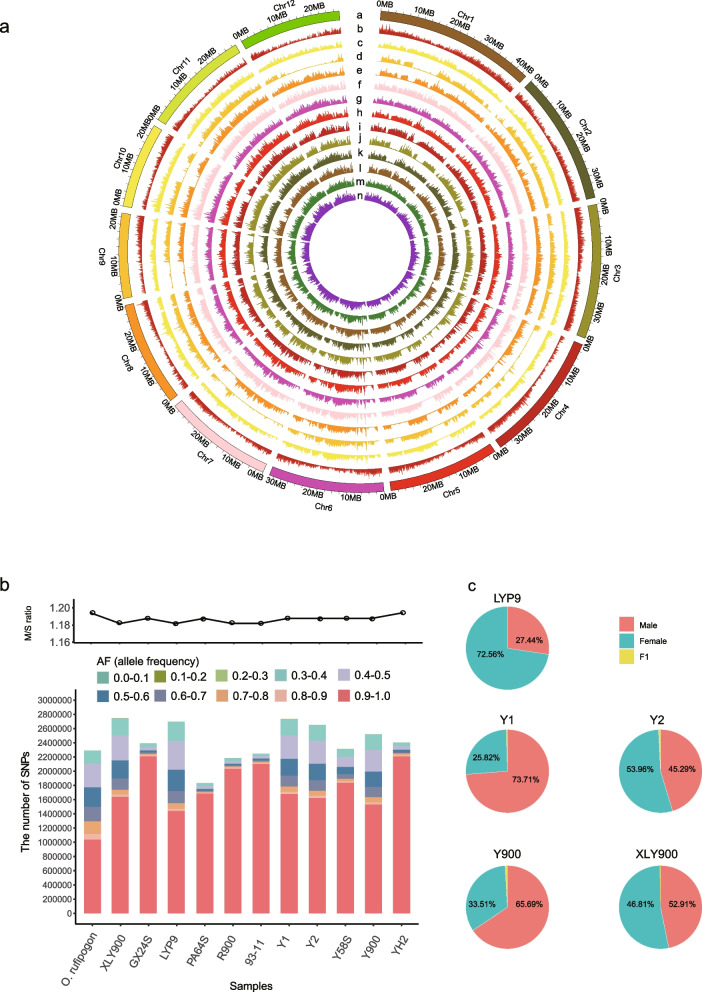
Genomic variants landscape of five super-hybrid rice and their hybrid progenitors. **a** Circos plot of SNP density within the genomes for the five super-hybrid and their hybrid progenitors, mapped in 1000 kb sliding windows. The outermost ring (*a*) represents 12 rice chromosomes, marked in Mb units. The second ring (*b*) illustrates gene density. Rings (*c–n*) represent SNP densities for rice varieties: LYP9, PA64S, Y58S, 93-11, Y1, Y2, YH2, R900, Y900, GX24S, XLY900, and *O. rufipogon*. **b** The top graph shows the ratio of non-synonymous to synonymous mutations, indicating the functional impact of genetic variation. The bottom bar chart displays the allele frequency (AF) distribution across different frequency spectra for the studied rice samples, revealing the polymorphism landscape. **c** Pie charts depict the genotype composition of the super-hybrid rice varieties in comparison to their parental progenitors, illustrating the inheritance pattern and genetic contribution from each parent

Super-hybrid rice exhibits strong stress resistance and the potential for increased yield, underscoring the importance of genomic features to comprehend the genetic traits of super-hybrid rice. In this study, we selected five *indica* super-hybrid rice varieties, namely LYP9, Y1, Y2, Y900, and XLY900, each with different yield gradients. We also included the parental progenitors, Y58S, R900, Guanxiang 24S (GX24S), PA64S, Yuanhui 2 (YH2), totaling 11 varieties. We obtained genomic NGS reads amounting to 478.86 M (71.67 GB bases) and proceeded to analyze their genomic mutation profiles (see Methods). After filtering, our data exhibited a clean data rate of > 98% (approximately 470.86 M reads, 70.63 GB bases in total) with all samples achieving Q30 scores > 94% and mapping rates to the reference genome > 98%, indicating high data quality (Additional file 1: Table S10). Through genomic variant analysis, a total of 26,715,592 SNP loci, 5,327,825 InDel loci, and 175,504 SVs loci were identified among five rice hybrids and their parental progenitors (Fig. 4a, Additional file 1: Table S11), with most sites exhibiting an allele frequency > 0.9, and minor genomic variation differences between samples (Fig. 4a, Additional file 6: Fig. S5). We conducted a statistical analysis of the distribution of SNPs and SVs sites across genome regions, and the results indicate that the distribution trends across different samples’ genome regions are nearly consistent (Additional file 7: Fig. S6, Additional file 8: Fig. S7 and Additional file 1: Table S12). Our statistical analysis indicates that the M/S ratio in each sample was > 1 (Fig. 4b), although there were fluctuations across the samples. This fluctuation could be indicative of varying levels of positive selection pressure acting on different *indica* populations. The differences observed may be associated with the distinct breeding goals that have shaped these varieties over time, suggesting that modern breeding programs are having a significant influence on the evolutionary pressures experienced by these populations. This pattern suggests that there may be a differential contribution of maternal and paternal genomes to the genetic makeup of these hybrids, which could be linked to the specific breeding strategies and gene duplication events in these super-hybrids (Fig. 4c).

### Genetic background analysis reveals japonica ancestry in the five indica super-hybrid rice varieties and frequent recent genetic exchange between the indica and japonica subgroups gives rise to diverse hybrid rice varieties

**Fig. 5:**
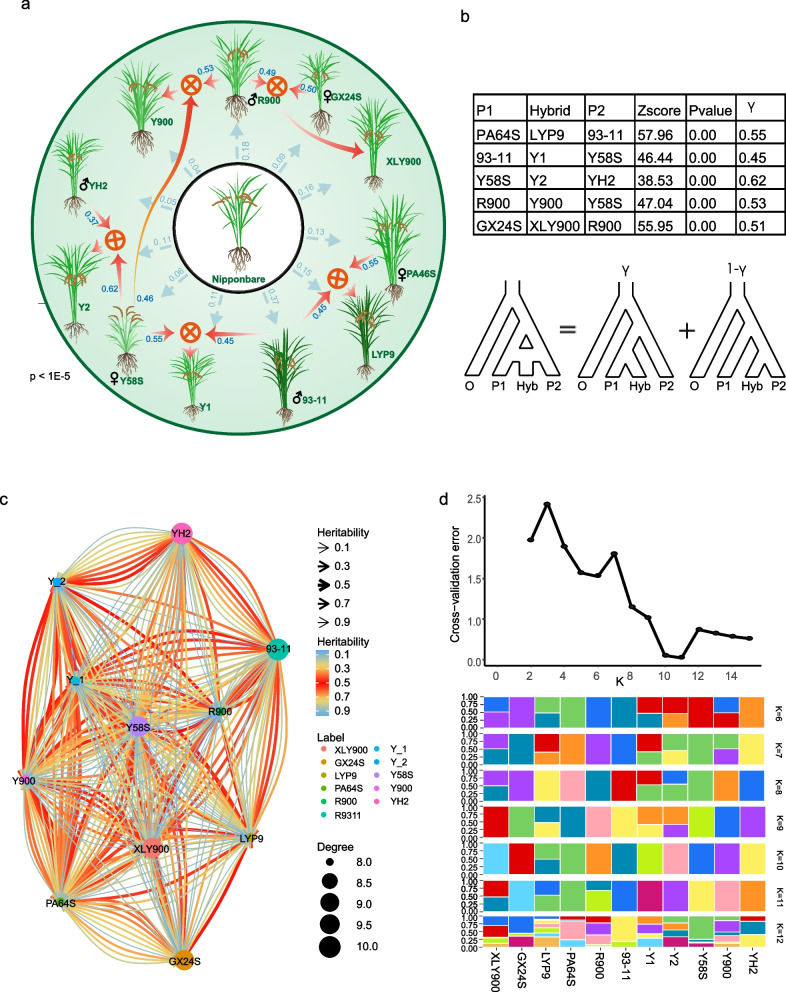
Analysis of hybridization history, population structure and genetic admixture in five elite super-hybrid rice varieties with their progenitors. **a** This circular diagram presents the hybridization history for each of five elite super-hybrid rice varieties as detected by HyDe, with significant hybridization events indicated where *P*-value < 0.05. The diagram highlights the complex interactions and genetic integration among the super-hybrids and their parental progenitors. **b** The table displays quantitative results from HyDe analysis, specifying the genetic proportions contributed by both parental progenitors to the super-hybrid *indica* rice varieties. The underneath accompanying schematic depicts the conceptual model for hybrid detection, representing hybrid (Hyb) as a composite of genetic input from two parental progenitors P1 (γ) and P2 (1 − γ), reflecting their respective heritability contributions. **c** The network graph illustrates the genetic heritability resulting from hybridization events among various rice varieties. Node sizes correspond to the degree of connectivity, while edge colors denote levels of heritability. Arrows point towards the hybrid offspring, indicating the direction of genetic contribution from the parents, with specific heritability values labeled alongside. **d** Top panel: cross-validation error plot to determine the optimal number of ancestral populations (K). Bottom panel: population admixture proportions visualized at the optimal K reveals the structural composition of genetic elements within each sample. The color coding represents unique genetic components derived from the ancestral populations

To delve deeper into the genetic history of *indica* super-hybrid rice, we performed genetic relationship analyses based on 90,113 high-quality SNPs in population samples from five super-hybrid rice and their progenitors. These were compared with *japonica* rice at the genomic level. HyDe analysis revealed that *indica* varieties inherited a degree of genetic material from *japonica* (Fig. 5a, Additional file 9: Fig. S8). The above phenomenon could be attributed to two factors: (1) Inheritance from parental varieties such as R900, PA64S, Y58S, and 93–11, which possess a *japonica* genetic background [30, 54] and (2) the introduction of the *japonica* genetic background is attributed to complex gene exchange between the *indica* and *japonica* subgroups, as highlighted in previous studies [42, 55]. Observation of the heritability of super-hybrid rice and its parents revealed that parental genetic contributions to hybrids in LYP9, Y1, Y2, XLY900, and other super rice predominantly originated from the maternal side. Y900, however, was the more influenced by paternal genetics. The *Z*-score for testing parental genetic contribution significance greatly exceeded ± 3 with a *P*-value approaching 0, indicating significant parental genetic influences (Fig. 5a, b). The trend of parental genetic sharing in all varieties, except Y1, is consistent with the conclusion of genomic mutation sharing between offspring and parents in the previous section (Figs. 5b, and 4c). The network genetic relationships constructed from the HyDe analysis (Additional file 1: Table S13) among all *indica* varieties revealed intricate genetic relationships within *indica* subspecies (Fig. 5c). In contrast, the admixture program [56] was used to analyze the genotype data of 90,113 SNPs, setting *K* from 3 to 15, to explore the genetic structure of these 11 *indica* rice varieties (Fig. 5d). By inferring the best *K* value, we identified two breaking points (*K* at 6 and 11). At *K* = 6, each super-hybrid rice possesses two ancestors with a mixing ratio of approximately 0.5, and the genetic composition of the super-hybrid rice is consistent with the parental selection in its breeding lineage. When *K* = 11, the cross-validation error is minimized, and since the population number is also 11, the estimated number of ancestors should be consistent with the population number. However, XLY900, R900, and LYP9 did not exhibit the characteristics of independent lineages, and the genetic composition of XLY900 included an unknown ancestor. This ambiguity likely results from limited sample sizes, reducing the statistical resolution to clearly define distinct ancestral populations.

In summary, the heritability and population structure analysis between hybrid rice and its parents vividly elucidate their complex hybridization history, consistent with the breeding history documented by breeders. Moreover, the recent signals of reciprocal hybridization between *indica* and *japonica* rice varieties explain the genetic factors underlying the formation of numerous elite hybrid varieties. This underscores the importance of deciphering more genetic resources in rice to guide the improvement of cultivated rice varieties.

### The prevalence of non-additive inheritance likely plays a major role in the manifestation of heterosis

**Fig. 6:**
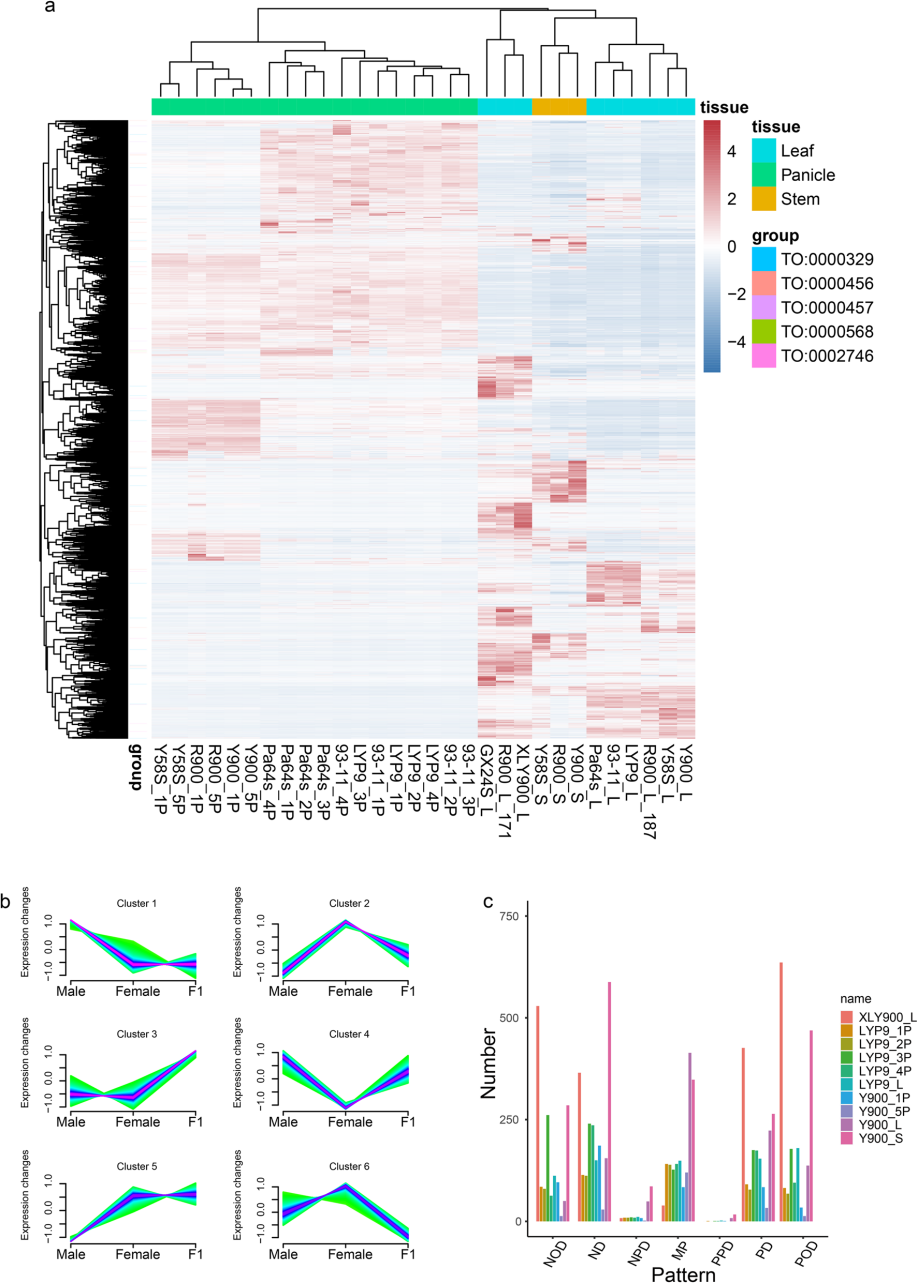
Gene expression profiling in elite super-hybrid rice varieties with their parental progenitors. **a** Heatmap illustrating the patterns of differentially expressed genes across various tissues in three super-hybrid rice varieties (LYP9, Y900, and XLY900) and their parental progenitors. The horizontal color bands represent different tissue types with corresponding expression transcripts per million (TPM) value, while vertical color bands classify genes according to Trait Ontology (TO: from China Rice Data Center). Expression levels are color-coded, with red indicating higher expression and blue indicating lower expression. **b** Line graphs depict the general expression trends across all differentially expressed genes within the three super-hybrid rice varieties and their parental lines, with separate trends plotted for male, female, and F_1_ hybrid tissues. **c** Bar graph categorizing the number of differentially expressed genes across the super-hybrid varieties, with color coding representing distinct patterns of gene expression in response to various biological and experimental conditions (“A” indicates additive gene, all others are non-additive)

Super-hybrid rice, renowned for its exceptional traits such as stress resistance and high yield, is pivotal to bolstering food security. To delve deeper into the formation of hybrid advantages in super-hybrid rice, we analyzed the transcriptome data of samples including LYP9, Y900, and XLY900 with their progenitors (Additional file 1: Table S14). We conducted pairwise comparisons for each super-hybrid rice variety with their parental progenitors, retaining genes with significant expression differences in any comparison group. The clustering of all differentially expressed genes unveiled a distinct tissue-specific trend across all samples (Fig. 6a, Additional file 1: Table S15). To enhance the observation of expression patterns between offspring and parents, we further employed Mfuzz [57] to cluster all genes based on their expression profiles across progenitors and offspring. This resulted in six clusters (Fig. 6b, Additional file 1: Table S16). Notably, clusters 3, 4, and 5 exhibited gene expression of hybrid offspring higher than one or both parents, suggesting that these highly expressed genes in offspring may contribute to trait superiority. Additionally, we classified genes based on their expression relationships between offspring and parents in different tissue samples of the LYP9, Y900, and XLY900 hybridization combinations (see Methods). The findings indicate that additive genes (A) account for approximately 25% in all offspring tissue samples of these three super-hybrid rice combinations, with non-additive genes predominating (Fig. 6c, Additional file 1: Table S17). A previous study on the LYP9 super-rice hybrid combination indicated that the predominance of non-additive genes may be a key factor in the formation of the trait dominance [41]. Gene annotation information related to yield traits can be found in Additional file 1: Table S18. Our study not only incorporates data from the LYP9 with its progenitors but also includes samples from the recently released Y900 and XLY900 super-hybrid rice varieties with their progenitors. The similar results observed in these super-hybrid rice varieties further underscore the significant role of non-additive expression genes in the formation of hybrid advantages in super rice.

### eQTL analysis

**Fig. 7:**
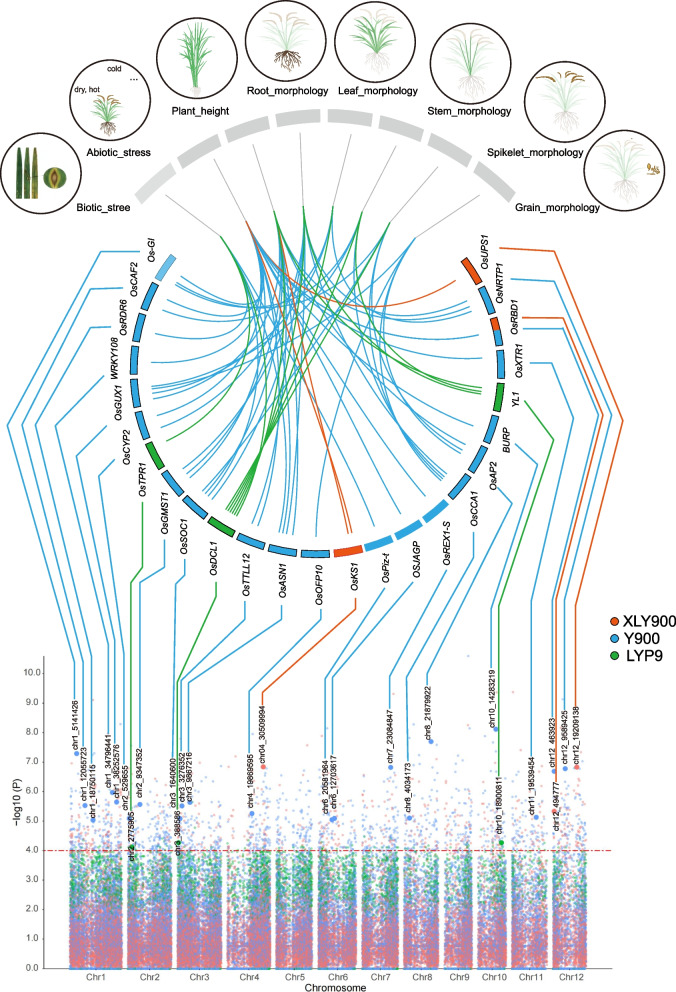
eQTL mapping of morphological traits in three elite super-hybrid rice varieties. Top panel: the Circos plot illustrates the association between morphological traits and linked candidate genes within significant SNP loci for the rice varieties XLY900, Y900, and LYP9. Traits such as plant height, root, leaf, stem, spikelet, and grain morphology are depicted in the outer ring with corresponding candidate gene connections drawn to the inner SNP locus ring. Bottom panel: A Manhattan plot presents a comprehensive view of SNP loci associated with the candidate genes for morphological traits across all rice chromosomes. Each dot represents an SNP, color-coded to distinguish the contributions from each of the elite super-hybrid rice varieties XLY900 (blue), Y900 (green), and LYP9 (red)

The influence of genomic mutations on gene regulation has been the subject of extensive research, with numerous studies underscoring their substantial impact [45, 58, 59]. Utilizing the FastQTL software, we correlated transcriptome expression data with genome mutation data for LYP9, Y900, and XLY900. We identified 82, 370, and 205 eQTL genes with high signal values in LYP9, Y900, and XLY900, respectively (LYP9: *P*-value < 1e − 4; Y900, XLY900: *P*-value < 1e − 5) (Additional file 1: Table S19). These genes included 26 known phenotype unique genes primarily associated with biotic and abiotic stresses, plant height, roots, stems, leaves, spikelet, and fruit morphology phenotypes (Fig. 7, Additional file 1: Table S20). For example, the *OsRBD1* gene can enhance the fungi yeast tolerance to abiotic stresses such as salt and osmotic stress by interacting with *OsSRO1a* gene [60]. In our study, the *OsRBD1* gene is a highly associated locus in Y900 and XLY900, especially in XLY900, a well-known saline-grown rice [61], this suggests the *OsRBD1* gene may plays a crucial role in salt tolerance. In comparison to the wild-type the *lfs* mutant showed a decrease in plant height and spike length, grain number per spike, 1000-grain weight and spikelet fertility [59]. Additionally, only 31 overlapping genes were identified between Y900 and XLY900 among these eQTL genes, with the remainder being specifically expressed in their respective samples (Additional file 10: Fig. S9).

To further investigate the potential molecular functions of eQTL loci across different hybridization combinations, we performed GO/KEGG enrichment analysis for the aforementioned eQTL genes. In GO enrichment analysis, the LYP9 hybridization combination group was mainly enriched to 31 GO terms (Additional file 11: Fig. S10, Additional file 1: Table S21), including 14 molecular functions (MF) and 17 biological processes (BP), predominantly concentrated in DNA-templated transcription (GO:0006351). The Y900 group was annotated to 98 GO categories (Additional file 11: Fig. S10, Additional file 1: Table S22), including 51 BP, 19 Cellular Components (CC), and 28 MF, primarily annotated on stress, defense responses to external (GO:0050896, GO:0006950, GO:0006952). XLY900 mainly included 36 BP, 14 CC, and 15 MF, predominantly enriched in metabolic processes (Additional file 11: Fig. S10, Additional file 1: Table S23). KEGG annotated 17 significant unique pathways, with LYP9, Y900, and XLY900 belonging to three, six, and ten, respectively (Additional file 12: Fig. S11, Additional file 1: Table S24). Notably, Y900 and XLY900 shared pathways for homologous recombination (ko03440) and nucleotide excision repair (ko03420) pathways.

### De novo SNP-associated genes are likely contributed to yield advantage formation in super-hybrid rice varieties

**Fig. 8:**
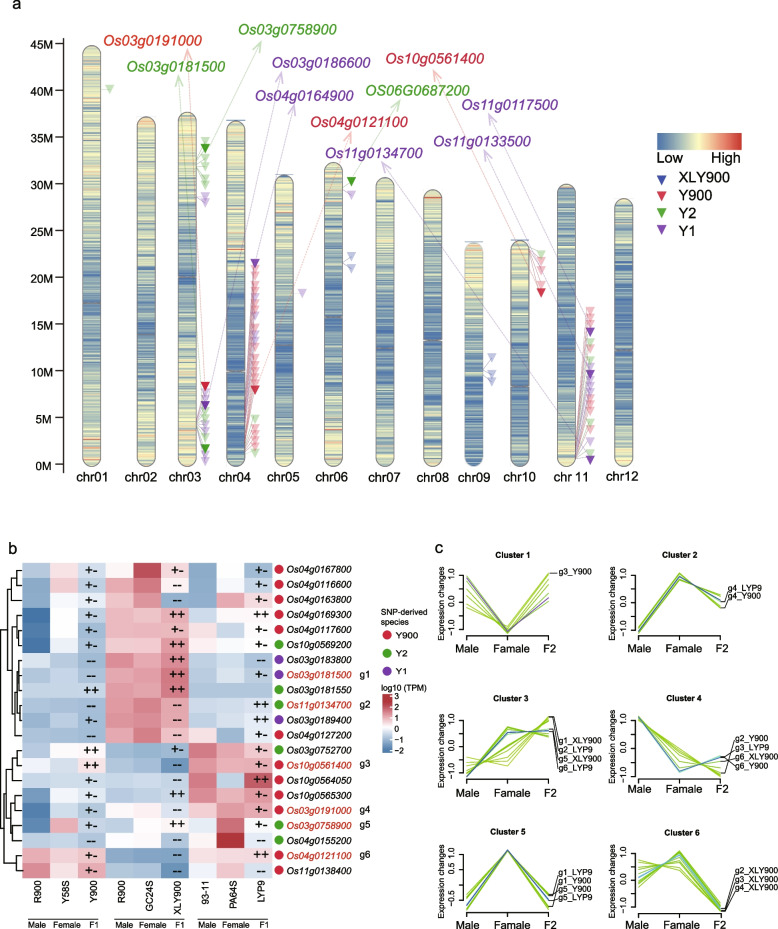
Distribution and expression pattern of genes with de novo SNPs identified for four elite super-hybrid rice varieties. **a** The graphic displays the density of genes across the 12 chromosomes of rice, with the color scale indicating gene density. Inverted triangles pinpoint the locations of genes harboring identified de novo SNPs, and genes with functional descriptions are labeled above each chromosome. **b** The heatmap details the differential expression levels of the genes identified in part (**a)** with genes with functional descriptions highlighted in red text. The colored scale on the right indicates the gene expression level, while the colored circle symbols next to gene names for super-hybrid rice varieties. The legend defines the symbols: “ + + ” indicates higher expression than both parents, “ + -” indicates higher expression than one parent, and “–” indicates lower expression than both parents. **c** The line charts represent the expression profiles of genes with functional descriptions identified in part (**b)** across male, female, and F_1_ hybrid samples from the super-hybrid rice combinations, with line colors corresponding to the gene come from different super-hybrid rice varieties

We hypothesized that de novo originated SNP sites (specific to offspring) might significantly contribute to the genetic basis of yield formation in high-yielding super rice. Across our analysis we identified 496 de novo SNP sites: 109 in Y1, 80 in Y2, 268 in Y900, 39 in XLY900. However, no de novo SNPs were observed in the offspring of LYP9. Of these, 103 sites associated with 81 genes were identified (Additional file 1: Table S25), including 11 markers linked to traits such as biotic and abiotic resistance, plant morphology, and other phenotypic (Fig. 8a, Additional file 1: Table S26). When correlating these 81 genes with the transcriptome data, only 21 genes were expressed in at least one sample of the three hybrid-rice combinations described above, including six marker genes (*Os03 g0191000*, *Os04 g0121100*, *Os11 g0134700*, *Os10 g0561400*, *Os03 g0181500*, and *Os03 g0758900*), showing varying expression trends across different hybrid rice combinations (Fig. 8b, c). Specifically, the heat tolerance gene *Os03 g0191000* exhibited lower expression in XLY900 compared to its parents [62]. *Os11 g0134700*, which exhibits diverse expression patterns under various biotic and abiotic stress conditions, such as drought, salinity, cold, and heat, and biotic stresses (including infection by *Magnaporthe oryzae*, and *Xanthomonas oryzae* pv. *oryzae* and *Rhizoctonia solani* nematodes) [63] had consistently lower expression levels than those of the parents in both Y900 and XLY900. The overexpression of *Os10 g0561400* may contribute to increased cold tolerance in Y900 [64]. The gene *Os03 g0181500* influences post-germination plant growth, with higher expression in LYP9 and XLY900 than in either parent [65]. The gene *Os03 g0758900*, which positively regulates the expression of disease-resistance genes, had an expression level higher than that of at least one parent in all three super rice combinations [66]. These genes may play a key role in the development of superior traits in super rice.

## Discussion

The origins of Asian rice domestication remain contentious, with prevailing hypothesis divided between a single domestication event and multiple independent domestications. Substantial research supports both hypotheses. Studies favoring a single origin tend to focus on specific genetic loci, whereas those advocating for multiple independent origins emphasize whole-genome variation. However, the intricate evolutionary history of Asian rice, further complicated by recent decades of breeding efforts [12], has resulted in complex genetic landscape among its subspecies. This complexity can significantly influence the accuracy of the subspecies origin hypotheses based on a limited number of genetic loci [67–69]. The recent availability of high-quality genomes of *Oryza* spp. facilitates the further elucidation of phylogenetic relationships within cultivated rices. Our study examined the complete gene set of 34 genomes, summarizing 39,984 multi-copy gene trees to construct a robust *Oryza* phylogeny. This approach minimized distortions caused by small gene scales and gene flow between certain subspecies, thereby circumventing misrepresentation of species relationships [70]. Consequently, our research contributes to the advancement of resolving the origins of Asian rice domestication. Our gene-level analysis across a broad range of *Oryza* spp. provides comprehensive evidence of the origins and domestications in Asian rice. Utilizing the species tree summarized by multi-copy gene family trees, we discovered that *indica* and *japonica* rice formed independent lineages with distinct *O. rufipogon* species as sister group, indicating separate origins from different *O. rufipogon* progenitors. Moreover, divergence time estimates at the ancestral node of *indica* and *japonica* indicated an earlier divergence of *japonica* relative to *indica*, which may be due to incomplete sampling of wild species in the species tree. Previous studies have established that Asian wild rice is divided into several varieties, and *O. nivara* also originates from *O. rufipogon* [67, 71]. Therefore, we speculate that there exists an unknown Asian wild rice ancestral species at the most common ancestor node of the *aus* and *indica* clades, so the actual divergence time 0.25 mya of this node is likely similar to that of the *japonica* rice in our study. In addition, the trend in genome-wide syntenic gene pair Ks distribution further corroborates that the age of two major subspecies origin is close to each other [11, 22, 23, 72]. Concurrently, analyses of genes within the nucleotide low-diversity region revealed parallel domestication times for the *indica* and *japonica* subspecies, thereby confirming the conclusion of independent domestication.

A previous study has suggested that *O. nivara* is a wild ancestor of the *aus* group [13]. By incorporating a more comprehensive array of wild rice species, we identified a close relationship between *aus* and the annual wild rice *O. nivara*, whereas *indica* was closer to the wild rice *O. rufipogon* w1654, with the *aus* and *indica* clades being sister groups. This evidence supports the independent origins of *aus*, *indica*, and *japonica* from different wild rice varieties, which is consistent with recent studies that have posited *O. nivara* as a separate lineage [11, 17, 73]. Notably, although previous studies suggested that *O. nivara* originated multiple times from different *O. rufipogon* populations [71, 73], our data describe *O. nivara* and *O. rufipogon* w1654 as members of the sister taxon (Fig. 1a). This may be due to under-sampling of our wild rice genome. Therefore, we hypothesized that *aus* and *indica* rice were differentiated from this unknown type of Asian wild rice, *O. rufipogon*.

Despite preponderance evidence supporting the polyphyletic origin of Asian rice, gene flow between Asian cultivated rice subspecies has also been observed, particularly in the introgressive influence of *japonica* on *indica* and its ancestors [22, 42, 50]. In our study, extensive recent genetic introgression from the *japonica* subgroup to the *indica* subgroup was observed (Figs. 2, and 5, and Additional file 9: Fig. S8), which is closely linked to modern breeding practices. Furthermore, the analysis of duplicate genes at the ancestral node of Asian rice (genes conforming to the ABAB pattern, indicating no hybridization between the MRCA of rice) further bolstered the hypothesis of independent origins of the *indica* and *japonica* subgroups. However, we noted a few gene duplications occurred in the MRCA of rice where genes were missing from either the *indica* or *japonica* branches, which we speculate may be due to the following: (1) different evolutionary patterns resulting in gene loss from certain subpopulations, (2) insufficient species coverage leading to undetected genes in certain subpopulations, (3) few gene flow events between subpopulations in the MRCA of *O. sativa*, especially from *japonica* to *indica* or *aus*, resulting in the absence of the gene in a particular branch of *indica* or *aus*. A prior study indicated that Asian wild rice is a hybrid swarm with extensive gene flow and feralization from domesticated rice, gene flow from domesticated rice to wild rice populations could result in genetic similarities between them [69], as observed at the *indica* rice ancestral node (Additional file 3: Fig. S2 d, f), where a large number of wild rice share some genetic background with cultivated rice that may also include gene flow from cultivated to wild rice. In addition, genetic introgression among wild rice is not restricted to Asian AA-genome species [51], but also occurs in African wild rice species and other genome types. These findings provide a more comprehensive explanation of the genetic background of Asian rice and contribute to the understanding of its diversity and evolutionary dynamics.

The continuous development of breeding technologies has led to significant improvements in super-hybrid rice. Research has shown an upward trend in yield for five super-hybrid rice combinations, LYP9, Y1, Y2, Y900, and XLY900, with XLY900 reached 18.0 t/hm^2^ [30, 74]. The successful breeding of these high-yielding varieties makes it possible to analyze their underlying genetic mechanisms. Genomic data analysis of these five hybrid combinations provides a comprehensive understanding of the genetic relationships and genomic characteristics of the progeny and parents. Allelic frequencies were generally high across different samples, with slight variations in the density distribution. These characteristic differences may be associated with positive selection acting on the relevant genomic regions of the rice varieties. Moreover, previous transcriptomic studies have suggested that non-additive effects inherently refer to the functional underperformance of two homozygous alleles from parents with insufficient background, whereas in the same background, one allele in the heterozygous F_1_ may fully function, exhibiting partial dominance, dominance, or even over-dominance [41]. Our findings that the transcriptomes of the super-hybrid rice combinations were distinctly tissue-specific and that a large number of alleles were expressed at higher levels in the hybrid progeny than in one or both of the parents, with a predominance of non-additive genes, led us to infer that they were associated with heterosis. This results in the expression of traits that are superior to those of the parents after crossing. Therefore, we hypothesize that this part of the gene may also play an important role in the formation of heterosis.

Numerous studies have confirmed the critical effect of genomic mutations on gene expression [45, 58, 59]. It has been demonstrated that phenotypic traits, such as hair color, shape, and thickness, are regulated by certain associated loci [44]. In our eQTL analysis, a group of stress-related genes significantly associated with mutant loci were identified. In addition, we found a large number of enriched GO terms in LYP9 related to environmental stress and defense, which illustrates the important role of these eQTL genes for LYP9 resistance traits. Furthermore, shared nucleotide excision repair and homologous recombination pathways in Y900 and XLY900 were observed in KEGG enrichment analysis. Previous research has indicated that DNA damage arises from both endogenous and exogenous factors [75]. Nucleotide excision repair is the primary pathway for removing DNA damage [76], and homologous recombination plays a crucial role in DNA repair and interchain crosslinking (ICL) [77]. These pathways were significantly enriched in Y900 and XLY900, whereas no relevant pathways were identified in LYP9. Therefore, we hypothesized that these factors above were closely associated with the resistance traits of Y900 and XLY900.

## Conclusions

The existence of gene flow between *indica* and *japonica* subspecies implies that they are not simply completely independent modes of domestication [42]. We conducted an analysis of Ka and Ks for 11 well-known rice domestication genes, as reported in previous studies [42, 78–80] between species within the *indica*, *japonica* subgroups and their recent common ancestral subspecies to characterize the domestication origin of these genes in *indica* and *japonica* subspecies (Additional file 4: Fig. S3). The results indicated that fluctuations in the Ka/Ks values of some genes and the domestication process have been subjected to varying degrees of positive selection. Some genes exhibited similar Ka and Ks values within subgroups but significant differences between them, such as *GW5* [81], *PROG1* [82], and *OsC1* [83], which were associated with grain shape, plant morphology, and pericarp luster. The Ks values of these genes were significantly higher in the *japonica* subpopulation than in the entire *indica* subpopulation, indicating that they originated and domesticated independently in the *indica* and *japonica* subpopulations, with *japonica* preceding *indica*. Some genes showed consistent Ka and Ks values in the *indica* and *japonica* subgroups, suggesting that they may have begun domestication in both subspecies simultaneously or as a result of gene flow during the early divergence of the subspecies, such as *SDR4* [84], *SH4* [85], *SHAT1* [86], and *Wx* [87], which were associated with seed dormancy, seed shattering, and glutinous endosperm. In summary, these results led us to hypothesize that subspecies formation occurred through different stages of evolution and that for some of the key traits, *japonica* began to be domesticated earlier than *indica*. In the subsequent history, in both subspecies, some traits were noticed and domesticated concurrently.

This study was designed to shed light on the origins of cultivated rice and the genomic characteristics underpinning the heterosis of super hybrid rice yield traits by integrating multi-omics data. By amalgamating the complete genomes of 33 high-quality Oryzeae species along with one outgroup *Brachypodium distachyon* genome, we constructed a robust phylogenetic relationship by summarizing 39,984 multi-copy gene trees. Our analysis revealed that *indica* and *japonica* as distinct lineages, supporting their independent origins from different wild rice species. Through assessing the collinear gene pairs and Ks distribution between cultivated rice subspecies and closed related wild rice varieties, as well as estimating the divergence time and Ks distribution of 54 putative domestication genes identified from nucleotide low-diversity regions, we found that although the overall domestication time of *indica* and *japonica* subspecies is similar. However, Ks analysis based on 11 key domestication genes revealed that the domestication time of some key genes related to morphology and grain shape in *indica* precedes that of *japonica* rice suggesting that the domestication of cultivated rice subspecies might have occurred in stages, with early domestication of certain traits in *japonica* rice, followed by simultaneous large-scale domestication of both *indica* and *japonica* rice to form modern subspecies. Analysis of the topological pattern of 1383 gene duplication in the MRCA of *O. sativa* did not support recent hybridizations between the MRCA of *indica* and *japonica* subspecies, further supporting their independent origins. Hybridization analyses, employing datasets of 1300 single-copy orthologous genes alongside 669 orthologous genes originating from gene duplication events in the MRCA of *Oryza sativa*, corroborate the hypothesis of ancient introgression from early divergent Oryzeae species contributing to rice speciation. This evidence, although limited, suggests a nuanced role of hybridization in the evolutionary origins of the MRCAs of *indica*, *japonica*, and *aus* subspecies. Genetic network analysis of whole-genome sequencing data from wild rice and cultivated rice subspecies, as well as analysis of hybridization, genetic networks, and population structure of whole-genome NGS data from five super rice combinations, revealed hybridization signals between *indica* and *japonica* rice and among *indica* rice subspecies, highlighting the complex breeding history of cultivated rice and the importance of introduction of cultivated rice subspecies for the breeding of elite varieties.

On the other hand, we conducted whole-genome NGS sequencing of five major hybrid *indica* rice varieties (LYP9, Y1, Y2, Y900, and XLY900) and their progenitors. Mutation analysis revealed genomic variation characteristics and different degrees of positive selection on those multiple hybrid-rice varieties. Hybridization detection and population genetics analysis strongly demonstrated extensive hybridization and introgression between the five commercialized super hybrid varieties and *indica* rice, indicating the integration of multiple genetic resources during the cultivation of super hybrid rice. Transcriptomic RNA-seq data analysis of three super hybrid varieties (XLY900, Y900, and LYP9) and their parental progenitors highlighted different gene expression patterns between hybrid varieties, emphasizing the potential importance of non-additive genes in trait superiority. eQTL analysis, integrating gene expression data with mutation maps, revealed that the resistance mechanisms of Y900 and XLY900 might be closely associated with shared recombination and DNA repair pathways. Additionally, it uncovered a set of genes that may influence the formation of advantageous traits.

In conclusion, our study provides comprehensive evidence to support the independent domestication hypothesis for the origins of the *indica* and *japonica* rice subspecies and their parallel domestication histories. Through analyses at different levels focusing on ancestral nodes of *indica* and *japonica* subspecies, we elucidate the potential impact of ancient genetic lineages on the formation of cultivated rice and the complex gene flow history among cultivated rice varieties. Additionally, analyses at the genomic and transcriptomic levels of super-hybrid rice provide new insights into the genetic basis of yield advantage and release a set of genes associated with superiority, thus providing data support for broadening breeding resources.

## Methods

### Experimental materials and data sources

The dataset for this investigation comprised coding sequences from 34 high-quality genomes, sourced from eminent public repositories: the Rice Genome Hub (https://rice-genome-hub.southgreen.fr), the National Genomics Data Center (https://ngdc.cncb.ac.cn/), and the National Center for Biotechnology Information (https://www.ncbi.nlm.nih.gov) (refer to Additional file 1: Table S2). For the construction of interspecific genetic networks, whole-genome sequencing datasets were retrieved from the European Nucleotide Archive (ENA, https://www.ebi.ac.uk/ena/browser/home), as documented in Additional file 1: Table S9, employing TreeMix v1.13 software [52] for interspecific genetic network analyses. Transcriptomic RNA-seq datasets includes tissue samples from roots, stems, and leaves of three elite super-hybrid rice varieties including Liangyoupei9 (LYP9), Y Liangyou 900 (Y900), and Xiang Liangyou 900 (XLY900) alongside their progenitor strains were curated from the NCBI Sequence Read Archive (https://www.ncbi.nlm.nih.gov/sra) and the CNCB database (https://ngdc.cncb.ac.cn/), as detailed in Additional file 1: Table S14. Five super-hybrid rice varieties including Liangyoupei9 (LYP9), Y Liangyou 1 (Y1), Y Liangyou 2 (Y2), Y Liangyou 900 (Y900), and Xiang Liangyou 900 (XLY900) were planted in Erlang Village, Jiusuo Town, Pingqiao District, Xinyang, Henan (114.08° E, 32.13° N), while their respective parents including Y58S, R900, Guanxiang 24S (GX24S), PA64SA, Yuanhui 2 (YH2) of the super-hybrid rice were cultivated in Jiaojiao Village, Jiusuo Town, Ledong County, Hainan Province (109.17° E, 18.73° N). Post-harvest, young foliar specimens were promptly submerged in liquid nitrogen for preservation and subsequently stored at − 80 °C. Genomic DNA was extracted, and high-throughput sequencing libraries were synthesized utilizing the Nextera DNA Flex Library Prep Kit (Illumina, San Diego, CA, USA). Subsequently, paired-end 150 sequencing is performed on the Illumina NovaSeq 6000 platform. In total, approximately 71.67 GB of bases was obtained.

### Genome-scale phylogenetic analysis with cultivated and wild rices

We curated a dataset comprising 33 high-quality genomes within the Oryzeae and *Brachypodium distachyon* serving as an outgroup, to refine the phylogenic resolution in subgroups level (Additional file 1: Table S2). The incorporation of coding sequences derived from the aforementioned genomes effectively minimize errors attributable to the disparate evolutionary trajectories of partial genes, which arise from varying degrees of bottleneck effects. The above approach also circumvents inconsistencies in outcomes that may result from partial gene flow between ancestral populations of cultivated rice originating from distinct sampling locations, as well as between cultivated rice and its ancestral groups. All annotated protein sequences from a total of 34 genomes were clustered into 39,984 orthologous groups (OGs) using OrthoFinder v2.5.5 [88] with default parameters. Protein sequences corresponding to each OG were extracted, and MAFFT v7.508 with parameter"–auto" [89] was performed for multiple sequence alignment. The protein alignments were converted into nucleotide multiple sequence sequences, guided by the original nucleotide coding sequences (CDS) by PAL2 NAL [90]. The maximum likelihood gene family trees were inferred using IQ-TREE v2.2.0.3 under the GTR + G model with 1000 bootstrap replicates [91]. Finally, all multi-copy gene family trees were summarized into a coalescent species tree using ASTRAL-Pro 2 (v1.13.1.3) [70] with default parameters.

### Molecular clock analysis

Molecular clock was used to estimate the divergence time within the Oryzoideae. On the one hand, we filtered out genes less than 900 bp and greater than 1200 bp in length from the 1300 single-copy gene clusters, resulting in 225 single-copy gene clusters used for species divergence time analysis. On the other hand, genes from 30 regions of low nucleotide diversity reported in a previous study [11] and genes related to yield collected from the China Rice Data Center (https://www.ricedata.cn/) were intersected; then, this set was intersected with 1300 single-copy gene clusters obtained from species phylogenomic analysis; finally, genes shorter than 900 bp and greater than 1200 bp were filtered out, resulting in 54 clusters of orthologous single-copy genes of putative domestication genes used to assess the domestication time among different subspecies. We extracted respectively the protein sequences of all the genes of these two gene clusters set using custom Perl scripts and then aligned using MAFFT v7.508 [89] for multiple sequence alignment. Subsequently, PAL2 NAL [90] was utilized to convert the alignments into CDS sequences. We concatenated the CDS sequences of the 225 gene clusters dataset and the 54-domestication gene dataset into separate sequence matrices, respectively. Then, we used MCMCtree [92] to infer the divergence time between species and the domestication time. Furthermore, to minimize potential systematic errors caused by multi-gene concatenation, MCMCtree was used to individually analyze the 54 putative domestication genes, and statistical analysis was conducted on the distribution of divergence node times to assess relative domestication timing.

### Ks analysis for syntenic and orthologous gene pairs to the divergence time of indica and japonica subspecies

The synonymous substitution rate (Ks) is typically not affected by selection pressure and can characterize the evolutionary history of species. First, we separately counted the Ks values of syntenic gene pairs between the Asian cultivated rice genomes and their corresponding MRCA species within their subgroups to assess the relative divergence time between species. Then, we assessed the genome duplication status by computing the Ks values of homologous gene pairs within the cultivated rice species itself. Homologous gene pairs were identified using DIAMOND v2.0.15.153 with parameters"-e 1e-10 –max-target-seqs 5 –masking 0 –no-self-hits –outfmt 6" [93]. Syntenic gene pairs were identified and analyzed using MCscanX [94] with parameters"-k 100 -s 5 -e 1e-10". The protein sequences of collinear gene pairs were aligned by MAFFT v7.508 with parameter"–auto" [89]. The protein alignments were converted into nucleotide alignments by PAL2 NAL [90]. Ks values were calculated for each aligned syntenic gene pairs using kaks_Calculator v2.0 with model NG [95]. Additionally, *aus*_N22, 93–11 and Nipponbare, which have better annotation quality, were used as representative species of *aus*, *indica* and *japonica* subspecies. For the 54 domestication gene clusters inferred from the previous molecular clock analyses in the regions of low nucleotide diversity, the genes of the representative species of subspecies *aus*, *indica*, and *japonica* in the gene clusters were used as orthologous gene pairs with the corresponding genes of the MRCA wild species of subspecies *aus*, *indica*, and *japonica*, and Ks analyses were carried out to assess the relative domestication times of the *aus*, *indica*, and *japonica* subspecies. In addition, for the more important 11 domesticated genes in rice [42, 78–80], Ks values were calculated between species within the *indica* and *japonica* subgroups, respectively, and the MRCA species of the subspecies to which they belong. All the above analyses were graphed using the R package ggplot2 [96].

### Phylogenomic profiling for detection of gene duplication events

Gene duplication events at each node of the species tree were identified by using Tree2GD v2.5 with parameters"–bp = 70 –sub_bp = 70 –species = 2 –root = MAX_MIX" [47]. Using the rooted species tree as a framework, all the gene family trees were rooted and mapping onto the species tree for tree reconciliation analysis, resulting in the identification of 1383 instances of gene duplication events. A detailed visualization of these gene duplication events, along with the corresponding alignments, is available at https://doi.org/10.6084/m9.figshare.27859797.v1. Subsequently, our custom Perl script was utilized to quantify the topological configurations of the gene trees implicated in these duplication events. A total of three topologies (ABAB, ABAX, and ABXB) of cultivated rice ancestor nodes were involved. The ABAB-type topologies suggest that subpopulation A and subpopulation B diverged independently at the ancestral node, whereas other types of topologies indicate potential gene flow between the two subpopulations.

### Identification of tandem gene duplication event

To investigate the origins of gene duplication events and determine whether such duplications in the ancestors of the genus *Oryza* were attributed to an allopolyploidy event, a comprehensive analysis was undertaken utilizing 93–11 (representative of the *indica* subspecies) and Nipponbare (representative of the *japonica* subspecies) as model organisms. This analysis focused on the quantification of tandem gene duplications, defined by the spatial proximity of duplicated gene pairs. Specifically, a criterion was employed wherein a maximum of ten intervening genes between each pair was permissible, thereby facilitating a detailed examination of the gene duplication landscape within these genomes.

### TE annotation and association analysis for duplicated genes and the presence of TEs

To elucidate the origins of gene duplication events and assess the hypothesis that the extensive gene duplications observed in the ancestors of the genus *Oryza* were not derived by allopolyploidization, we conducted a comprehensive TE annotation and association analysis. Specifically, the genomes of the *indica* and *japonica* rice representatives, Nipponbare and 93–11 respectively, were subjected to TE identification using the RepeatMasker v4.1.2 (https://www.repeatmasker.org/). The analysis employed parameters “-species rice -xsmall -s -norna -no_is -nolow -gff” and integrated the Dfam 3.8 [97] and Repbase databases (https://www.girinst.org/) for TE annotation. To explore the hypothesis that the observed extensive gene duplications may be mediated by TEs, we conducted a statistical analysis of TEs located within 2 kb upstream and downstream regions of duplicated genes compared to non-duplicated genes, based on the genomic annotation of both Nipponbare and 93–11. We utilized the chi-square test to statistically analyze the association between the presence of TEs and gene duplications, aiming to gain deeper insights into the role of TEs in the genome evolution of *Oryza* species.

### Identification and filtration of SNPs, and structural variations (SVs) by utilizing whole-genome sequencing data derived from super-hybrid rice and its parental progenitors

Genomic NGS data generated for the five super rice combinations in this study were used for genomic variant site analysis. The specific steps are as follows: Firstly, the raw data were filtered and quality controlled using Trimmomatic (v0.39, parameters: LEADING:10 TRAILING:10 SLIDINGWINDOW:4:20 MINLEN:36) [98] to obtain high-quality reads and high-quality reads were aligned to the Nipponbare genome using BWA software (0.7.1) [99] mem module to generate SAM format files. SAM files were sorted into BAM files using SAMtools v1.16.1 [100] and duplicate sequences were marked using the Picard software MarkDuplicate module. After that, GVCF files for each sample were generated using GATK v4.3 with the HaplotypeCaller module [101], and variant sites were obtained using GenotypeGVCFs, with SNP/Indel sites obtained using SelectVariants. Finally, low-quality SNPs were filtered using VariantFiltration (QD < 10.0 || FS > 60.0 || MQ < 40.0 || SOR > 3.0 || MQRanksum < −12.5 || ReadPosRanksum < −8.0) and these sites underwent annotation through the utilization of SnpEff software [102]. Considering the issues of insufficient coverage for low depth and amplification of sequencing errors for extremely high depth, sites with less than one-third of the average depth and more than three times the average depth were filtered out. In addition, structural variant analyses were performed. Using the LUMPY program [103] and Manta software [104] with default parameters, the BAM files aligned to the Nipponbare reference genome were processed. Subsequently, SURVIVOR [105] was employed to merge the results and calculate different types of structural variation features. The density plot of genome-wide variant including SNPs and SVs from elite super-hybrid rice and its parental progenitors were visualized by Circos v0.69–8 [106] with 1-Mb sliding windows.

### Hybridization and introgression analysis among Oryza species

To elucidate gene flow dynamics within rice species and its interactions with wild rice species, we conducted hybridization analyses at both gene and SNPs level. At gene level, we employed a rigorous approach to identify putative single copy orthologs from an extensive dataset of 39,984 rooted multi-copy gene trees by using module Ortho_Retriever from PhyloTracer (https://github.com/YiyongZhao/PhyloTracer). To reduce the potential biases introduced by gene loss, we meticulously curated a gene dataset comprising single-copy gene sets with 100% species coverage across 34 genomic datasets. To investigate the signals of ancient hybridization among the MRCAs of the three-rice subspecies, *indica*, *japonica*, and *aus*, our study utilized 1383 gene duplicates that originated from the MRCA of rice for subsequent HyDe analyses. The module Ortho_Retriever from PhyloTracer was employed to split these multi-copy gene trees into single-copy gene trees and to pinpoint the orthologous genes. Ultimately, we identified a total of 669 orthologous genes that originated from the MRCA of rice, each exhibiting a minimum taxa coverage of 50%. We separately processed these two sets of genes as follows. First, the protein sequences from these orthologous gene sets were aligned by MAFFT v7.508 [89] with parameters"–auto". These protein alignments were then converted into CDS alignments with the guidance from nucleotide alignments by using PAL2 NAL [90]. The resulting CDS alignments were then concatenated into a supermatrix alignment for hybridization analysis by HyDe [50], operating under default parameters with a significance threshold of *P*-value < 0.05.

In the SNP-level analysis, we conducted genetic analysis between Asian rice species and within *indica* rice using two datasets. The first dataset referred to as dataset 1, comprised genomic NGS data from previous studies, including Asian cultivated rice and wild rice (Additional file 1: Table S9). The second dataset, referred to as Dataset 2, consisted of genomic NGS data from this study, including LYP9, Y1, Y2, Y900, and XLY900, along with their parental lines. The steps were as follows: Firstly, the raw data were filtered and quality-controlled using Trimmomatic and high-quality reads were aligned to the Nipponbare genome using BWA-MEM software to generate SAM format files. SAMtools v1.16.1 converted the SAM files as BAM files and tagged for repetitive sequences using the Picard software MarkDuplicate module to mark duplicate sequences. After that, GVCF files were generated for each sample using GATK v4.3 and combine all GVCF files using the “CombineGVCFs” parameter to obtain the variant sites using GenotypeGVCFs and SNP sites using SelectVariants. For these SNP loci, we used VariantFiltration to filter low-quality SNPs, and given that anomalous depths can lead to a widening of the data error, we filtered loci below one-third of the mean depth and above three times the mean depth. Finally, Filter loci with missing data > 0.2 and filter linkage disequilibrium (LD) loci using PLINK with the parameter"–indep-pairwise 50 10 0.2" [107] to obtain high-quality SNPs. We concatenated the SNPs (Dataset 1: 35,502; Dataset 2: 90,113) generated from these two datasets into sequence matrices, respectively, and performed hybridization and introgression analyses using HyDe software [50]. In addition, due to the existence of datasets that do not contain all types of Asian wild rice species, we used different strategies for our analyses. For dataset 1, we performed genetic network analyses between subspecies using TreeMix v1.13 with parameter"-k 500" [52]. For dataset 2, we performed intra-subspecies genetic network analyses using HyDe.

### Population structure analysis by ADMIXTURE

To analyze the genealogical structure of super rice at the level of genomic mutations, we performed population structure analysis using ADMIXTURE [108] for 90,113 high-quality SNP loci identified in the genomic NGS data of the five samples of super rice and its parents in the present study, with a K-value ranging from 2 to 15. The results were presented graphically using the ggplot2 software package for R. The optimal K values and admixture proportions were visualized using the ggplot2 package in R.

### Gene expression quantification and clustering for RNA-seq data

RNA-seq data collected for the three super-hybrid rice varieties (LYP9, Y900, and XLY900) and their hybrid progenitors, tissues including roots, stems, leaves, and panicle fluff, are detailed in Additional file 1: Table S12. High-quality reads were obtained by filtering and quality control of raw data using Trimmomatic (v0.39, options: LEADING:10 TRAILING:10 SLIDINGWINDOW:4:20 MINLEN:36) [98]. The clean reads were then mapped to the *O. sativa* Nipponbare reference genome using HISAT2 (v2.2.1) [109], followed by quantification of gene expression using StringTie [110]. To preserve more information regarding differentially expressed genes, for each gene in the hybrid combinations, comparisons were made between offspring and both parental lines. If a gene exhibited significant differential expression in any comparison, it was retained and resulting in a final set of differentially expressed genes. The dataset was clustered using the R package ComplexHeatmap [111]. To facilitate the discovery of gene expression pattern between progeny and parents, all genes were organized into data matrices according to parental combinations and clustering was conducted using the R package Mfuzz [57].

### Classification for gene expression patten between progeny and progenitors

Previous studies on LYP9 suggest that non-additive genes may be the dominant factor in dominance formation. To further validate the measurement point of view, we additionally included the transcriptome data of two super rice combinations, Y900 and XLY900, to classify the genes statistically with consistent criteria [41]. Genes were classified as follows: If the A gene was significantly higher than the higher parent gene, the gene was classified as positive overdominance (POD). If the A gene was significantly lower than the lower parent gene, the gene was classified as negative overdominance (NOD). If the A gene was significantly higher than the middle-parent (The middle-parent expression level is calculated as (P1 + P2)/2, where P1 and P2 represent the expression levels of each gene in both parents.) but showed no significance from the higher parent, the gene was classified as positive dominance (PD). If the A gene was significantly lower than the middle-parent but showed no significance from the lower parent, the gene was classified as negative dominance (ND). If the A gene was significantly higher than the middle-parent and significantly lower than the higher parent, the gene was classified as positive partial dominance (PPD). If the A gene was significantly lower than that of the middle-parent and significantly higher than that of the lower parent, the gene was classified as negative partial dominance (NPD). If the A gene was not significantly different from the middle-parent, significantly lower than the higher parent, and significantly higher than the lower parent, the gene was classified as additive expression gene (A).

### eQTL analysis

In our study, we conducted a linear association analysis to investigate expression quantitative trait loci (eQTLs) across three elite super-hybrid rice varieties (LYP9, Y900, and XLY900) and their hybrid parents, integrating comprehensive gene expression data from their transcriptomes with genomic variant information. Using FastQTL software [112], we identified significant eQTL sites by applying a significance threshold of *P* ≤ 1e − 5 for Y900 and XLY900, and a relaxed threshold of *P* ≤ 1e − 4 for LYP9 (exhibited a lower number of associated sites) to accommodate the identification of high-value signal sites. This analysis revealed 25 genes associated with significant eQTL sites, implicated in a range of biological functions from stress responses to the determination of key plant morphological traits. To elucidate the relationships between phenotypes, genes, and eQTL sites, we generated detailed association diagrams using the ggplot2 package in R, providing a visual representation of the genetic architecture underlying trait variation in these rice varieties.

### KEGG and GO enrichment analysis

We conducted gene tree reconciliation with the species tree across 20 rice genomes to identify gene duplication events. This analysis uncovered a total of 1383 gene duplication events in the common ancestor of cultivated rice (*Oryza sativa*) and its wild progenitor (*Oryza rufipogon*), involving 24,916 genes. To explore the functional implications of these duplicated genes, we performed Gene Ontology (GO) and Kyoto Encyclopedia of Genes and Genomes (KEGG) enrichment analyses. Annotation files derived from the 20 genomes served as the background dataset, and functional annotations were assigned using EggNOG-mapper [48]. This comprehensive approach enabled robust enrichment analyses, providing valuable insights into the biological processes and pathways associated with the duplicated genes and emphasizing their functional significance in rice evolution. In addition, we obtained a group of genes significantly associated with the mutant loci in the eQTL analysis. For these above two gene sets, Kyoto Encyclopedia of Genes and Genomes (KEGG) and Gene Ontology (GO) enrichment analysis was performed using the OmicShare tool (https://www.omicshare.com/tools/home/report/koenrich.html).

### Identification of de novo SNPs in elite super-hybrid rices

The methodology for identifying shared genetic sites between progeny and parental genotypes involved treating each hybrid and their progenitors as a discrete unit. Within this framework, a genetic site in the progeny was classified as homozygous for a mutation if it matched the genotype of either parent, indicating inheritance from that parent. Sites consistent with both parents’ genotypes were categorized as shared, whereas sites where the mutant genotype of progeny diverged from both parents were designated as de novo. These sites underwent annotation through the utilization of SnpEff software [102] to delineate genes harboring de novo SNPs. Concurrently, gene expression profiles were ascertained by integrating transcriptome data, facilitating a comprehensive understanding of the genetic and functional landscape of these elite super-hybrid rice varieties.

## Supplementary Information

Additional file 1: Table S1. Comparative characteristics of various hybrid rice varieties. Table S2. Information on the genomic datasets employed for phylogeny reconstruction, encompassing 34 genomes including 33 genomes from Oryzeae and *Brachypodium distachyon* as outgroup. Table S3. Distribution of gene duplication types in ancestral nodes of cultivated rice, focusing on tandem duplication, genomic collinearity, and other duplication forms, based on *O. sativa* Nipponbare (*japonica*) and *O. sativa* 93–11 (*indica*). Table S4. A chi-square test was conducted comparing transposable elements (TEs) annotated in the *japonica* representative Nipponbare and the *indica* representative 93–11 against duplicated genes. TE-associated genes were defined as those located within 2 kb upstream or downstream of TE regions. Table S5. GO enrichment analysis results (Q-value < 0.05) for 24,916 genes from 1,383 gene duplications originating from the MRCA of *Oryza sativa* in Fig. 1, based on 20 genomes using the OmicShare cloud platform (https://www.omicshare.com/). Table S6. KEGG pathway enrichment analysis results (Q-value < 0.05) for 24,916 genes from 1,383 gene duplications originating from the MRCA of *Oryza sativa*, based on 20 genomes using the OmicShare cloud platform (https://www.omicshare.com/). Table S7. Summary of divergence time estimation of 54 putative domesticated genes. These genes were identified in 30 regions of genomic low nucleotide diversity. The MRCA of *O. sativa*, *japonica*, *indica*, and *aus* in the table correspond to nodes of the species tree in Fig. 1, the time unit: million years ago (Mya). Table S8. Summary of Ks (synonymous substitution rate) values for 54 orthologous gene pairs of putatively domesticated genes, identified across 30 genomic regions exhibiting low nucleotide diversity. To approximate the onset of domestication process for various cultivated rice ancestors, we employed multiple representative genomes from different *Oryza* subgroups. This was done to calculate the Ks values for those 54 orthologous domesticated genes, thereby providing an estimation of the origin of domestication process. The domestication origin of MRCA of *O. sativa* was inferred from a comparison between *O. rufipogon* w1943 and *O. rufipogon* w1654. The domestication origin of MRCA of *japonica* is inferred from a comparison between *O. rufipogon* w1943 and *O. sativa* Nipponbare. The domestication origin of the MRCA of *indica* was inferred from a comparison between *O. rufipogon* w1654 and *O. sativa* 93–11. Lastly, the domestication origin of the MRCA of *aus* was inferred from a comparison between *O. nivara* and *O. sativa aus* N22. Table S9: Metadata of whole-genome sequencing datasets obtained from ENA used in TreeMix analysis. Table S10. Detailed quality assessment of newly sequenced whole-genome data preprocessing results for five super-hybrid rice varieties and their parental progenitors in this study. Table S11. Quantification of SNPs, InDels, and SVs detected in newly sequenced whole-genome data for five super-hybrid rice varieties with their parental progenitors and *Oryza rufipogon* in this study. Table S12. Quantification on the classification of genomic variants and their distribution in different rice varieties from newly sequenced whole genome sequencing data in this study. Table S13. Summary of heritability estimates for five super rice hybrids, their parental lines, and Nipponbare. This analysis is based on hybrid data derived from 90,113 SNP loci, where P1 and P2 denote the maternal and paternal parents, respectively, and Gamma represents the fraction of genetic contribution from P1 to the hybrid. Table S14. The information of RNA sequencing data for the three super-hybrid rice varieties (LYP9, Y900, and XLY900) with their parental progenitors. Table S15. Summary of gene expression profiles across three super-hybrid rice varieties and their progenitors. This table presents a comprehensive overview of gene expression data collected from three super-hybrid rice varieties: LYP9, Y900, and XLY900. It also includes data from their progenitors: GX24S, PA64 s, R900, 93–11, and Y58S. The data encompasses gene expression levels in different plant tissues, specifically leaves, stems, and panicles, offering insights into the gene expression dynamics across various stages of plant growth and development in both the hybrid varieties and their ancestral lines. Table S16. Differential gene expression clustering in super-hybrid rice varieties and their progenitors. This table delineates the results of gene expression clustering using the MFUZZ algorithm for three super-hybrid rice varieties, namely LYP9, Y900, and XLY900, along with their progenitor strains. Displayed within the table are clusters of differentially expressed genes. Each gene's expression data is associated with a specific tissue sample, using a naming convention that includes the gene identifier, variety code, and tissue type, separated by underscores. Table S17. Summary of the gene expression patterns in various tissue samples.'M_'indicates genes expressed from the paternal side,'F_'designates maternal gene expression, and'F_1__*'highlights the gene expression in the hybrid progeny. The patterns of gene expression are coded as follows: POD for positive overdominance, NOD for negative overdominance, PD for positive dominance, ND for negative dominance, PPD for positive partial dominance, NPD for negative partial dominance, and A for additive expression. For comprehensive definitions of these terms, readers are directed to consult the Methods section of the document. Table S18. Summary of annotation information for genes related to yield traits from the China Rice Data Center database (https://www.ricedata.cn/). Table S19. Summary of eQTL genes identified for three super-hybrid rice varieties and their progenitors. This table provides a compilation of eQTL genes that have been identified in three super-hybrid rice varieties, namely LYP9, Y900 and XLY900, employing a significance threshold of P-value < 1e-5, and for LYP9 with a relaxed threshold of P-value < 1e-4. Table S20. Trait ontology annotations for marker genes with significant eQTL signals. This table provides detailed trait ontology annotations for marker genes that exhibit strong eQTL signals, aiding in the elucidation of genetic influences on specific traits. The associated trait information for each gene was sourced from The Rice Annotation Project (RAP, http://rice.uga.edu/). Table S21. Gene Ontology (GO) enrichment analysis for eQTL genes specifically expressed in LYP9. Table S22. GO enrichment analysis for eQTL genes specifically expressed in Y900. Table S23. GO enrichment analysis for eQTL genes specifically expressed in XLY900. Table S24. KEGG pathway enrichment analysis of eQTL genes in three super-hybrid rice varieties (LYP9, Y900, and XLY900). Table S25. Summary of yield-related genes linked to de novo SNP loci in four super-hybrid rice varieties. This table compiles a list of yield-related genes that are associated with de novo single nucleotide polymorphism (SNP) loci identified in four super-hybrid rice varieties: Y1, Y2, Y900, and XLY900. Notably, these specific SNP loci have not been detected in the LYP9 variety. Table S26. Characterization of trait-associated genes with de novo SNP loci and expression profiles. This table outlines the traits of genes linked with de novo SNP (single nucleotide polymorphism) loci as indicated in Fig. 8a, detailing the gene IDs, gene names, chromosomal positions, gene start and end points, and trait descriptions. Genes that are highly expressed are highlighted in red. Additionally, the table includes information on where these genes are expressed across three super-hybrid rice varieties LYP9, Y900, and XLY900.

Additional file 2: Fig. S1. A summary of the relative domestication time between subspecies of cultivated rice and the distribution characteristics of Ks density of all homologous gene pairs within the cultivated rice species. (a) Boxplots showing the Ks values of all syntenic gene pairs between the wild rice and the representative species of two subspecies of cultivated rice, respectively, which are boxplot representations of the corresponding species in Fig. 1d. (b) shows the trend in Ks density for all paralogous gene pairs in the respective genomes of *Oryza sativa* rice varieties, and the right-hand legend shows population information. (c) A molecular clock analysis based on concatenated sequences of the 54 putative domestication genes identified from low nucleotide diversity regions, illustrating the relative timing of domestication across subspecies of cultivated rice. The blue bars represent the 95% highest posterior density (HPD) for the estimated divergence times. The pentagrams represent the MRCA of each rice subgroup, with divergence times denoted to the right. The number of gene duplication events is indicated at the upper left of each node, with the red star marking the MRCA of *Oryza sativa*.

Additional file 3: Fig. S2. Genomic visualization of gene duplications originating from the MRCA of *Oryza sativa* by Circos and dot plots. This figure delineates the gene duplication dynamics within the MRCA of *Oryza sativa* by using *japonica* (Nipponbare, left panels) and *indica* (93–11, right panels) subpopulations as representatives. Subfigures (a) and (b) display the collinearity among duplicated genes across the rice genome. Subfigures (c) and (d) present a genome-wide mapping of synonymous substitution rates (Ks) for the Nipponbare and 93–11 genome, indicating the absence of recent whole-genome duplication (WGD) events in the ancestors of genus *Oryza*. Subfigures (e) and (f) illustrate the chromosomal distribution of duplicated gene pairs within subspecies of the Asian cultivated rice, highlighting the positional distribution of genomic regions of gene duplication events, 402 and 81 duplicated gene pairs were involved in Nipponbare and 93–11, respectively.

Additional file 4: Fig. S3. Comparative analysis of Ka, Ks, and Ka/Ks ratios for 11 key domestication genes in *japonica* and *indica* subspecies. This figure summarizes the non-synonymous (Ka), synonymous (Ks), and the ratio of non-synonymous to synonymous substitutions (Ka/Ks) for eleven crucial genes associated with the domestication of *japonica* and *indica* rice subspecies. The histogram subpanels represent the variation in these values across the *japonica* and *indica* comparisons with different wild and cultivated rice varieties. Each bar color correlates with a specific comparison as indicated in the legend, facilitating the assessment of divergence and evolutionary pressures exerted on these domestication-related genes.

Additional file 5: Fig. S4. Hybridization and introgression signal for the MRCAs of *O. sativa* and three rice subspecies, respectively. The heatmap displays hybridization signals as detected by HyDe for each MRCAs of *Oryza sativa*, *indica*, *japonica*, and *aus*, respectively, annotated with red-highlighted branches on the cladogram to denote the detected clade. The analysis was designed to incorporate two distinct gene sets of single-copy gene. The first gene set, consisting of 669 genes, covering 50% of Asian rice taxa; These genes have been identified as originating from duplication events in the MRCA of *O. sativa*, and the purpose for this analysis is to analyze the ancient hybridization among different MRCAs of *indica*, *japonica* and *aus* (a, c, e and g). The other gene set comprised 1,300 orthologous genes covering 100% rice taxa (b, d, f and h). These two gene sets collectively facilitate hybridization analyses of the ancestral hybridization among different rice subspecies, as well as providing insights into the more recent genetic hybridization events. See methods for detailed gene selection procedures. Subfigures (a) and (b) focus on the progenitors of Asian cultivated rice, whereas (c) and (d) concentrate on the *indica* lineage, (e) and (f) on the *japonica* lineage, and (g) and (h) on the *aus* lineage. Each small colored square on the heatmap corresponds to a hybridization signal identified by HyDe, with the color intensity representing the probability of inheritance for the associated taxa on the y-axis. Contiguous squares of identical shading that compose a larger block within the heatmap, along with their corresponding taxa on the axes forming a monophyletic clade, which is indicated with a node number on a green background.

Additional file 6: Fig. S5. Genomic SVs landscape of five super-hybrid rice and their hybrid progenitors. The subfigures (a-e) depict the landscape of structural variations for deletion, duplication, inversion, insertion, and translocation, respectively. The Circos plots represent the SV density within the genomes of five super-hybrid rice varieties and their progenitors, mapped using a 1000 kb sliding window approach. The outermost ring (a) represents 12 rice chromosomes, marked in Mb units. The second ring (b) illustrates gene density. Rings (c-n) represent SV densities for the rice varieties: LYP9, PA64S, Y58S, 93–11, Y1, Y2, YH2, R900, Y900, GX24S, XLY900, and *O. rufipogon*, respectively.

Additional file 7: Fig. S6. Distribution of genomic variations (SNPs and InDels) in different genomic regions of five super-hybrid rice varieties and their parental progenitors. The figure illustrates the distribution of SNPs and InDels across various genomic regions; specifically, 2 kilobases (kb) upstream and downstream of genes, exons, introns, and non-coding regions in five super-hybrid rice varieties alongside their respective hybrid parents. Panel (a) quantifies the ratio of SNPs, while panel (b) details the ratio of InDels, both of which are critical indicators of genetic diversity and evolution within these varieties. The data are represented as stacked bar graphs, with color coding to differentiate between the genomic regions.

Additional file 8: Fig. S7. Distribution of SVs in genomic regions of five super-hybrid rice varieties and their parental progenitors. Panel a and b of Fig. S7 depict the distribution of SVs, including deletions (DEL), duplications (DUP), insertions (INS), inversions (INV), and translocations (TRA), across different genomic regions of five super-hybrid rice varieties and their respective parental progenitors. These regions encompass two kilobases (kb) upstream and downstream of genes, as well as exons, introns, and intergenic regions. The data are represented in stacked bar charts for each sample, with colors indicating the type of SVs.

Additional file 9: Fig. S8. Summary of the hybridization signal (γ) from HyDe analysis among the five super-hybrid rice varieties and their parental progenitors. Heatmap displays the hybridization signals detected by HyDe among five sets of super-hybrid rice and their parental progenitors, as well as the Nipponbare, with *O. rufipogon* as an outgroup. Each colored square on the heatmap corresponds to a hybridization signal identified by HyDe, with the intensity of color representing the genetic probability of the associated taxa on the Y-axis.

Additional file 10: Fig. S9. A Venn diagram summarizing overlapping eQTL loci among three super-rice varieties (LYP9, Y900, and XLY900) and their parental progenitors.

Additional file 11: Fig. S10. The bubble plot summarizing the biological processes of eQTL genes for three super-hybrid rice varieties and their parental progenitors based on Gene Ontology (GO). The figure summarizes the annotation of important biological processes (BP) terms (P-value < 0.05) for eQTL genes in the LYP9, Y900, and XLY900 super-hybrid rice varieties and their parental progenitors based on the Gene Ontology (GO) database.

Additional file 12: Fig. S11. The bubble plot summarizes the KEGG pathway annotations (P-value < 0.05) for eQTL genes within the LYP9, Y900, and XLY900 super-hybrid rice varieties and their parental progenitors.

## Data Availability

The genomic sequencing data generated for all samples from the super rice combinations in this study have been deposited in the Sequence Read Archive (SRA) database at the National Center for Biotechnology Information (NCBI), United States. The datasets can be accessed through BioProject under accession number PRJNA924010 [113]. Multiple sequence alignments (FASTA format) and PDF visualizations of 1383 gene duplication events identified in the common ancestor of cultivated rice (*Oryza sativa*) and its wild progenitor (*Oryza rufipogon*) across 20 genomes are publicly available on Figshare: 10.6084/m9.figshare.27859797.v1 [114]. The following publicly available genome assembly were included for comparative genomic analyses: *Oryza alta* [115]; *Oryza barthii* [51]; *Oryza brachyantha* [51]; *Oryza coarctata* [116]; *Oryza glaberrima* [51]; *Oryza glumaepatula* [51]; *Oryza glumipatula* [51]; *Oryza granulata* [117]; *Oryza longistaminata* [51]; *Oryza meridionalis* [51]; *Oryza nivara* [51]; *Oryza punctata* [51]; *Oryza rufipogon* w1654; *Oryza rufipogon* w1943 [118]; *Oryza sativa* aus N22 [51]; *Oryza sativa* basmati Basmati334 [119]; *Oryza sativa* basmati Domsufid [119]; *Oryza sativa indica* 93–11 [51]; *Oryza sativa indica* IR8 [51]; *Oryza sativa indica* MH63 [120]; *Oryza sativa indica* R498 [121]; *Oryza sativa indica* R900 [55]; *Oryza sativa indica* Y58S [55]; *Oryza sativa indica* ZS97 [120]; *Oryza sativa japonica* Kitaake [122]; *Oryza sativa japonica* subtrop Chao meo [123]; *Oryza sativa japonica* trop1 azucena [123]; *Oryza sativa japonica* trop2 Ketan Nangka [123]; *Oryza sativa* L. Nipponbare [124]; *Oryza sativa* Nerica4; *Oryza sativa* Nerica L19; *Leersia perrieri* [51]; *Zizania latifolia* [125]; *Brachypodium distachyon* [3] (Fig. 1a). Further details and data download links are provided in Additional file 1: Table S2.

## Abbreviations

BWA: Burrows-Wheeler aligner
CDS: Coding sequence
CNCB: China national center for bioinformation
Diamond: Double index alignment of next-generation sequencing data
ENA: European nucleotide archive
eQTL: Expression quantitative trait locus
GATK: Genome analysis toolkit
GD: Gene duplication
GO: Gene ontology
GVCF: Genomic variant call format
KEGG: Kyoto Encyclopedia of Genes and Genomes
MAFFT: Multiple sequence alignment with fast Fourier transform
MRCA: Most recent common ancestor
NCBI: National center for biotechnology information
ND: Negative dominance
NGS: Next-generation sequencing
NOD: Negative overdominance
NPD: Negative partial dominance
PD: Positive dominance
POD: Positive overdominance
PPD: Positive partial dominance
RNA-seq: RNA sequencing
SNP: Single-nucleotide polymorphism
SRA: Sequence read archive
