# Supplementary material for: The origin and evolution of cultivated rice and genomic signatures of heterosis for yield traits in super-hybrid rice
Source: BMC Biol. 2025 Jun 4;23:153. doi: 10.1186/s12915-025-02255-2 (PMC12139199; doi:10.1186/s12915-025-02255-2)
Supplement: Supplementary file 2 — Additional file 2: Fig. S1. A summary of the relative domestication time between subspecies of cultivated rice and the distribution characteristics of Ks density of all homologous gene pairs within the cultivated rice species. (a) Boxplots showing the Ks values of all syntenic gene pairs between the wild rice and the representative species of two subspecies of cultivated rice, respectively, which are boxplot representations of the corresponding species in Fig. 1d. (b) shows the trend in Ks density for all paralogous gene pairs in the respective genomes of Oryza sativa rice varieties, and the right-hand legend shows population information. (c) A molecular clock analysis based on concatenated sequences of the 54 putative domestication genes identified from low nucleotide diversity regions, illustrating the relative timing of domestication across subspecies of cultivated rice. The blue bars represent the 95% highest posterior density (HPD) for the estimated divergence times. The pentagrams represent the MRCA of each rice subgroup, with divergence times denoted to the right. The number of gene duplication events is indicated at the upper left of each node, with the red star marking the MRCA of Oryza sativa. [file 12915_2025_2255_MOESM2_ESM.pdf]

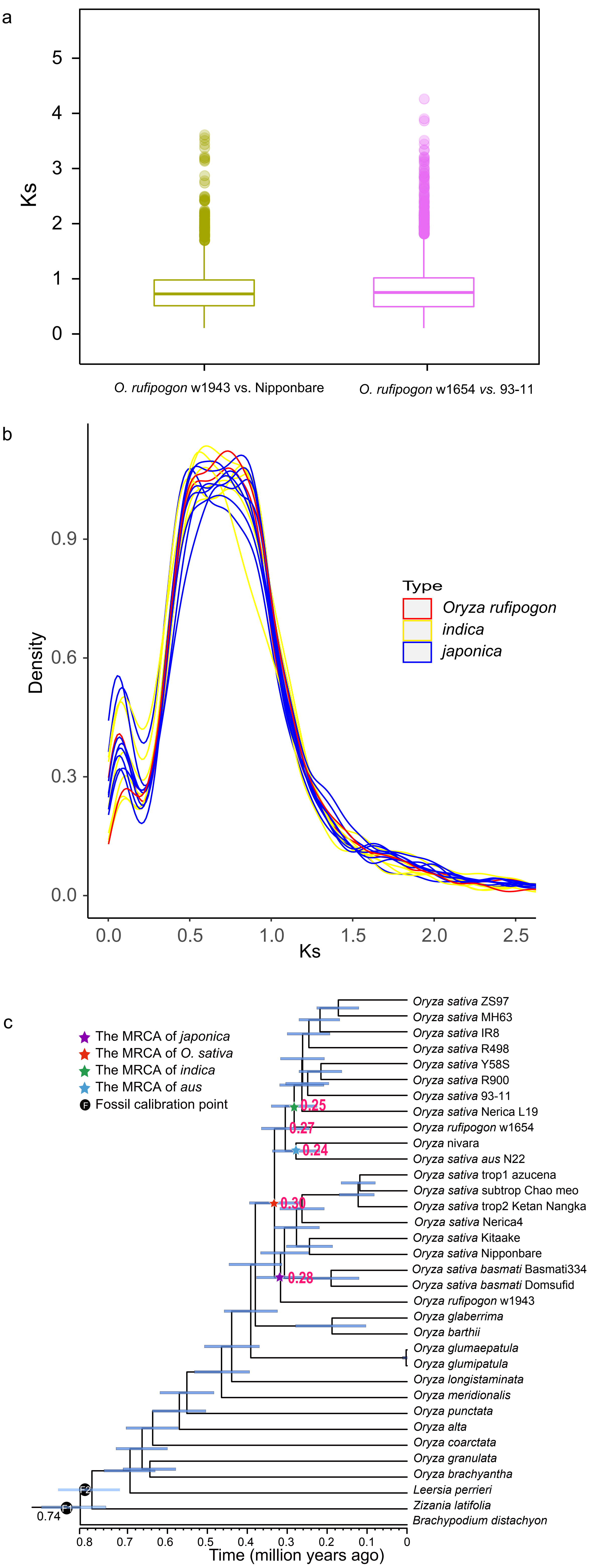

Figure S1. A summary of the relative domestication time between subspecies of cultivated rice and the distribution characteristics of Ks density of all homologous gene pairs within the cultivated rice species.
(a) Boxplots showing the Ks values of all syntenic gene pairs between the wild rice and the representative species of two subspecies of cultivated rice, respectively, which are boxplot representations of the corresponding species in Fig. 1d. (b) shows the trend in Ks density for all paralogous gene pairs in the respective genomes of *Oryza sativa* rice varieties, and the right-hand legend shows population information. (c) A molecular clock analysis based on concatenated sequences of the 54 putative domestication genes identified from low nucleotide diversity regions, illustrating the relative timing of domestication across subspecies of cultivated rice. The blue bars represent the 95% highest posterior density (HPD) for the estimated divergence times. The pentagrams represent the MRCA of each rice subgroup, with divergence times denoted to the right. The number of gene duplication events is indicated at the upper left of each node, with the red star marking the MRCA of *Oryza sativa*.
