# Supplementary material for: The origin and evolution of cultivated rice and genomic signatures of heterosis for yield traits in super-hybrid rice
Source: BMC Biol. 2025 Jun 4;23:153. doi: 10.1186/s12915-025-02255-2 (PMC12139199; doi:10.1186/s12915-025-02255-2)
Supplement: Supplementary file 3 — Additional file 3: Fig. S2. Genomic visualization of gene duplications originating from the MRCA of Oryza sativa by Circos and dot plots. This figure delineates the gene duplication dynamics within the MRCA of Oryza sativa by using japonica (Nipponbare, left panels) and indica (93–11, right panels) subpopulations as representatives. Subfigures (a) and (b) display the collinearity among duplicated genes across the rice genome. Subfigures (c) and (d) present a genome-wide mapping of synonymous substitution rates (Ks) for the Nipponbare and 93–11 genome, indicating the absence of recent whole-genome duplication (WGD) events in the ancestors of genus Oryza. Subfigures (e) and (f) illustrate the chromosomal distribution of duplicated gene pairs within subspecies of the Asian cultivated rice, highlighting the positional distribution of genomic regions of gene duplication events, 402 and 81 duplicated gene pairs were involved in Nipponbare and 93–11, respectively. [file 12915_2025_2255_MOESM3_ESM.pdf]

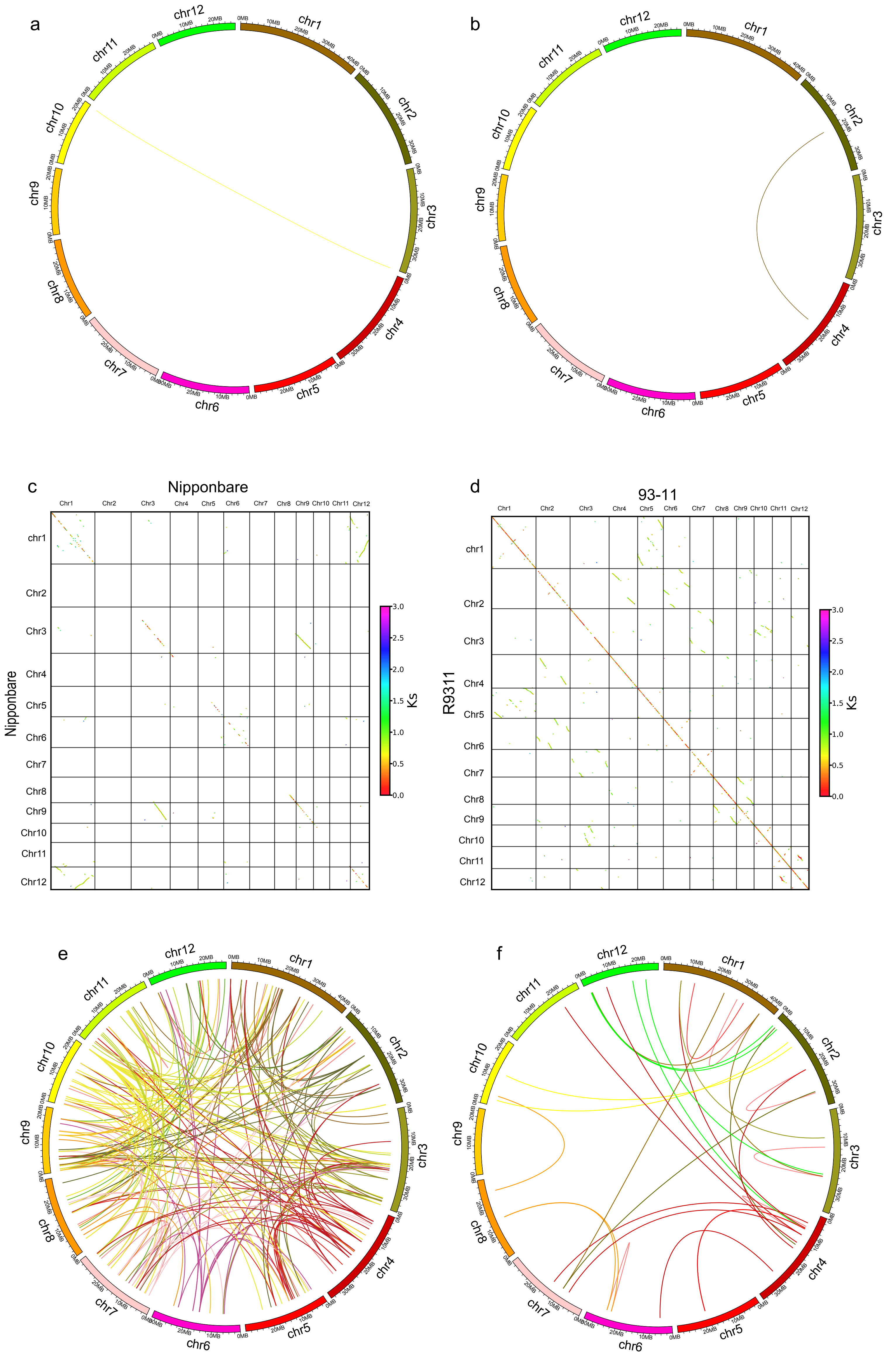

Figure S2. Genomic visualization of gene duplications originating from the MRCA of *Oryza sativa* by Circos and dot plots.

This figure delineates the gene duplication dynamics within the MRCA of *Oryza sativa* by using *japonica* (Nipponbare, left panels) and *indica* (93-11, right panels) subpopulations as representatives. Subfigures (a) and (b) display the collinearity among duplicated genes across the rice genome. Subfigures (c) and (d) present a genome-wide mapping of synonymous substitution rates (Ks) for the Nipponbare and 93-11 genome, indicating the absence of recent whole-genome duplication (WGD) events in the ancestors of genus *Oryza*. Subfigures (e) and (f) illustrate the chromosomal distribution of duplicated gene pairs within subspecies of the Asian cultivated rice, highlighting the positional distribution of genomic regions of gene duplication events, 402 and 81 duplicated gene pairs were involved in Nipponbare and 93-11, respectively.
