# Supplementary material for: The origin and evolution of cultivated rice and genomic signatures of heterosis for yield traits in super-hybrid rice
Source: BMC Biol. 2025 Jun 4;23:153. doi: 10.1186/s12915-025-02255-2 (PMC12139199; doi:10.1186/s12915-025-02255-2)
Supplement: Supplementary file 4 — Additional file 4: Fig. S3. Comparative analysis of Ka, Ks, and Ka/Ks ratios for 11 key domestication genes in japonica and indica subspecies. This figure summarizes the non-synonymous (Ka), synonymous (Ks), and the ratio of non-synonymous to synonymous substitutions (Ka/Ks) for eleven crucial genes associated with the domestication of japonica and indica rice subspecies. The histogram subpanels represent the variation in these values across the japonica and indica comparisons with different wild and cultivated rice varieties. Each bar color correlates with a specific comparison as indicated in the legend, facilitating the assessment of divergence and evolutionary pressures exerted on these domestication-related genes. [file 12915_2025_2255_MOESM4_ESM.pdf]

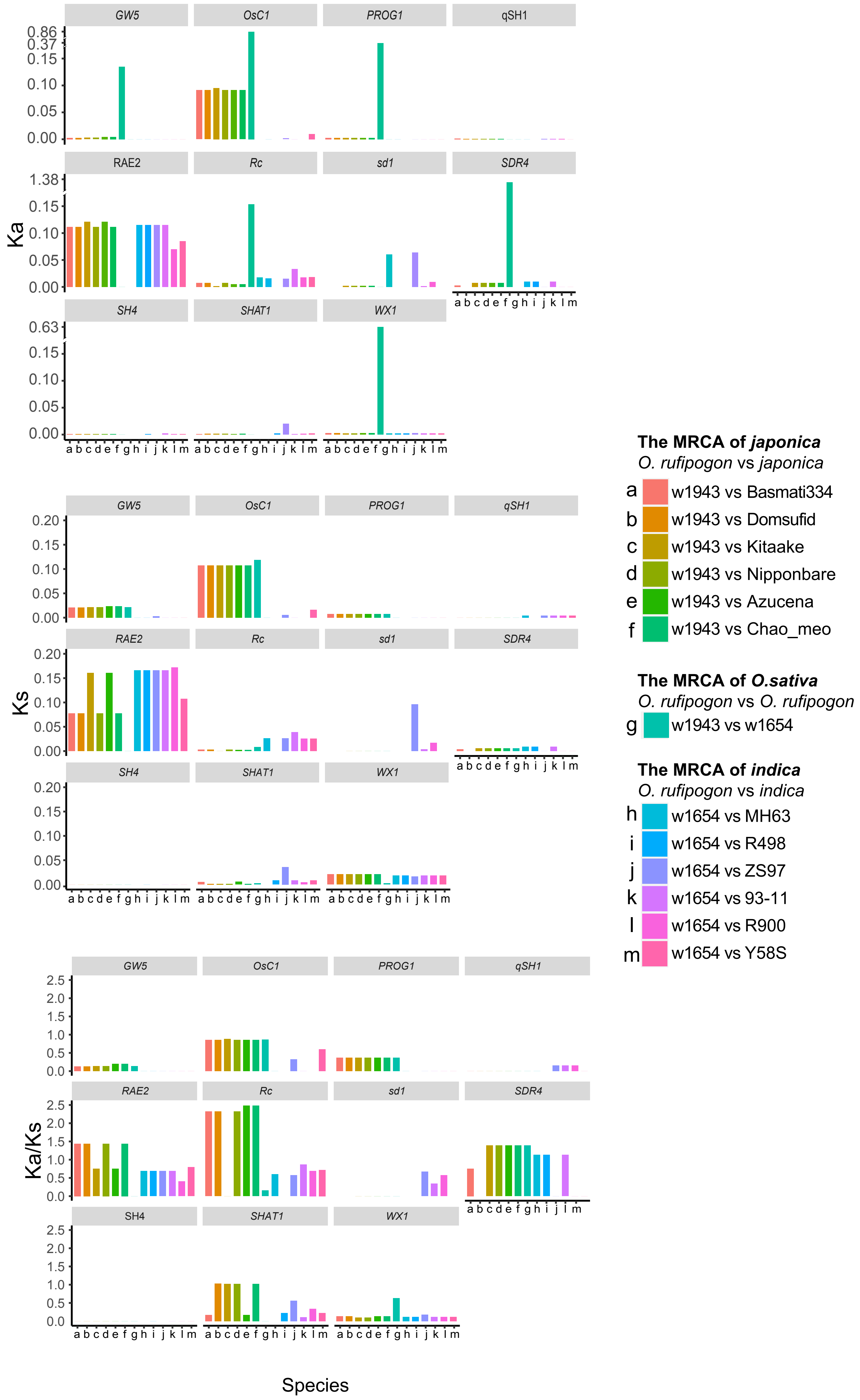

Figure S3. Comparative analysis of Ka, Ks, and Ka/Ks ratios for 11 key domestication genes in *japonica* and *indica* subspecies.

This figure summarizes the non-synonymous (Ka), synonymous (Ks), and the ratio of non-synonymous to synonymous substitutions (Ka/Ks) for eleven crucial genes associated with the domestication of *japonica* and *indica* rice subspecies. The histogram subpanels represent the variation in these values across the *japonica* and *indica* comparisons with different wild and cultivated rice varieties. Each bar color correlates with a specific comparison as indicated in the legend, facilitating the assessment of divergence and evolutionary pressures exerted on these domestication-related genes.
