# Supplementary material for: The origin and evolution of cultivated rice and genomic signatures of heterosis for yield traits in super-hybrid rice
Source: BMC Biol. 2025 Jun 4;23:153. doi: 10.1186/s12915-025-02255-2 (PMC12139199; doi:10.1186/s12915-025-02255-2)
Supplement: Supplementary file 5 — Additional file 5: Fig. S4. Hybridization and introgression signal for the MRCAs of O. sativa and three rice subspecies, respectively. The heatmap displays hybridization signals as detected by HyDe for each MRCAs of Oryza sativa, indica, japonica, and aus, respectively, annotated with red-highlighted branches on the cladogram to denote the detected clade. The analysis was designed to incorporate two distinct gene sets of single-copy gene. The first gene set, consisting of 669 genes, covering 50% of Asian rice taxa; These genes have been identified as originating from duplication events in the MRCA of O. sativa, and the purpose for this analysis is to analyze the ancient hybridization among different MRCAs of indica, japonica and aus (a, c, e and g). The other gene set comprised 1,300 orthologous genes covering 100% rice taxa (b, d, f and h). These two gene sets collectively facilitate hybridization analyses of the ancestral hybridization among different rice subspecies, as well as providing insights into the more recent genetic hybridization events. See methods for detailed gene selection procedures. Subfigures (a) and (b) focus on the progenitors of Asian cultivated rice, whereas (c) and (d) concentrate on the indica lineage, (e) and (f) on the japonica lineage, and (g) and (h) on the aus lineage. Each small colored square on the heatmap corresponds to a hybridization signal identified by HyDe, with the color intensity representing the probability of inheritance for the associated taxa on the y-axis. Contiguous squares of identical shading that compose a larger block within the heatmap, along with their corresponding taxa on the axes forming a monophyletic clade, which is indicated with a node number on a green background. [file 12915_2025_2255_MOESM5_ESM.pdf]

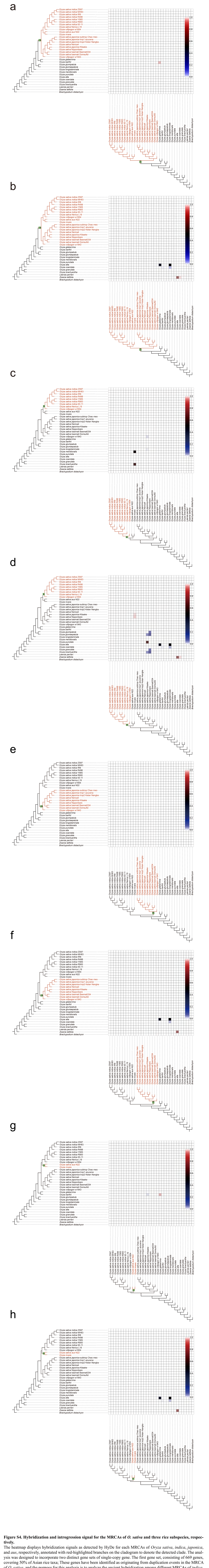

Figure S4. Hybridization and introgression signal for the MRCAs of *O. sativa* and three rice subspecies, respectively.
The heatmap displays hybridization signals as detected by HyDe for each MRCAs of *Oryza sativa*, *indica*, *japonica*, and *aus*, respectively, annotated with red-highlighted branches on the cladogram to denote the detected clade. The analysis was designed to incorporate two distinct gene sets of single-copy gene. The first gene set, consisting of 669 genes, covering 50% of Asian rice taxa; These genes have been identified as originating from duplication events in the MRCA of *O. sativa*, and the purpose for this analysis is to analyze the ancient hybridization among different MRCAs of *indica*, *japonica* and *aus* (a, c, e and g). The other gene set comprised 1,300 orthologous genes covering 100% rice taxa (b, d, f and h). These two gene set collectively facilitate hybridization analyses of the ancestral hybridization among different rice subspecies, as well as providing insights into the more recent genetic hybridization events. See methods for detailed gene selection procedures. Subfigures (a) and (b) focus on the progenitors of Asian cultivated rice, whereas (c) and (d) concentrate on the *indica* lineage, (e) and (f) on the *japonica* lineage, and (g) and (h) on the *aus* lineage. Each small colored square on the heatmap corresponds to a hybridization signal identified by HyDe, with the color intensity representing the probability of inheritance for the associated taxa on the y-axis. Contiguous squares of identical shading that compose a larger block within the heatmap, along with their corresponding taxa on the axes forming a monophyletic clade, which is indicated with a node number on a green background.
