# Supplementary material for: The origin and evolution of cultivated rice and genomic signatures of heterosis for yield traits in super-hybrid rice
Source: BMC Biol. 2025 Jun 4;23:153. doi: 10.1186/s12915-025-02255-2 (PMC12139199; doi:10.1186/s12915-025-02255-2)
Supplement: Supplementary file 6 — Additional file 6: Fig. S5. Genomic SVs landscape of five super-hybrid rice and their hybrid progenitors. The subfigures (a-e) depict the landscape of structural variations for deletion, duplication, inversion, insertion, and translocation, respectively. The Circos plots represent the SV density within the genomes of five super-hybrid rice varieties and their progenitors, mapped using a 1000 kb sliding window approach. The outermost ring (a) represents 12 rice chromosomes, marked in Mb units. The second ring (b) illustrates gene density. Rings (c-n) represent SV densities for the rice varieties: LYP9, PA64S, Y58S, 93–11, Y1, Y2, YH2, R900, Y900, GX24S, XLY900, and O. rufipogon, respectively. [file 12915_2025_2255_MOESM6_ESM.pdf]

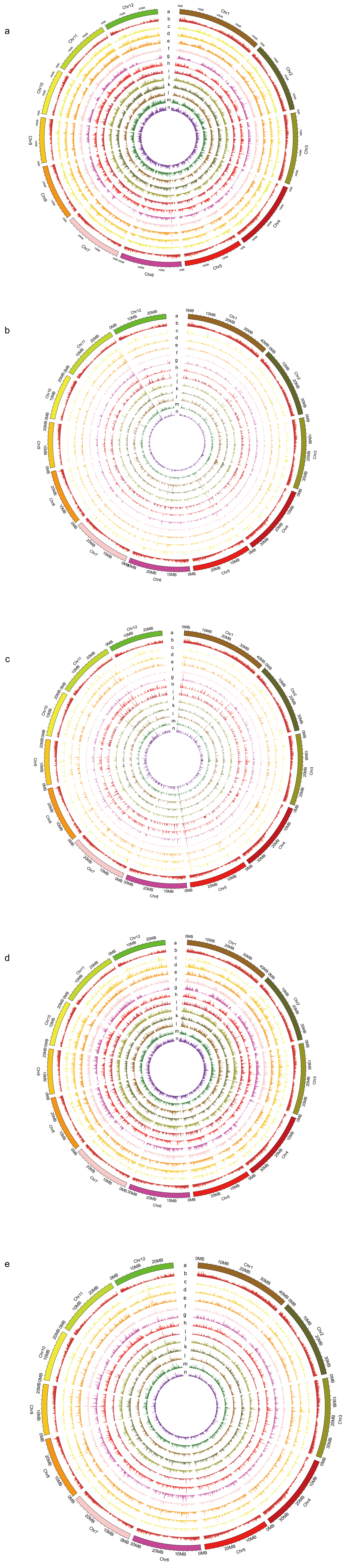

Figure S5. Genomic SVs landscape of five super-hybrid rice and their hybrid progenitors.
The subfigures (a-e) depict the landscape of structural variations for deletion, duplication, inversion, insertion, and translocation, respectively. The Circos plots represent the SV density within the genomes of five super-hybrid rice varieties and their progenitors, mapped using a 1000 kb sliding window approach. The outermost ring (a) represents 12 rice chromosomes, marked in Mb units. The second ring (b) illustrates gene density. Rings (c-n) represent SV densities for the rice varieties: LYP9, PA64S, Y58S, 93-11, Y1, Y2, YH2, R900, Y900, GX24S, XLY900, and *O. rufipogon*, respectively.
