# Supplementary material for: The origin and evolution of cultivated rice and genomic signatures of heterosis for yield traits in super-hybrid rice
Source: BMC Biol. 2025 Jun 4;23:153. doi: 10.1186/s12915-025-02255-2 (PMC12139199; doi:10.1186/s12915-025-02255-2)
Supplement: Supplementary file 7 — Additional file 7: Fig. S6. Distribution of genomic variations (SNPs and InDels) in different genomic regions of five super-hybrid rice varieties and their parental progenitors. The figure illustrates the distribution of SNPs and InDels across various genomic regions; specifically, 2 kilobases (kb) upstream and downstream of genes, exons, introns, and non-coding regions in five super-hybrid rice varieties alongside their respective hybrid parents. Panel (a) quantifies the ratio of SNPs, while panel (b) details the ratio of InDels, both of which are critical indicators of genetic diversity and evolution within these varieties. The data are represented as stacked bar graphs, with color coding to differentiate between the genomic regions. [file 12915_2025_2255_MOESM7_ESM.pdf]

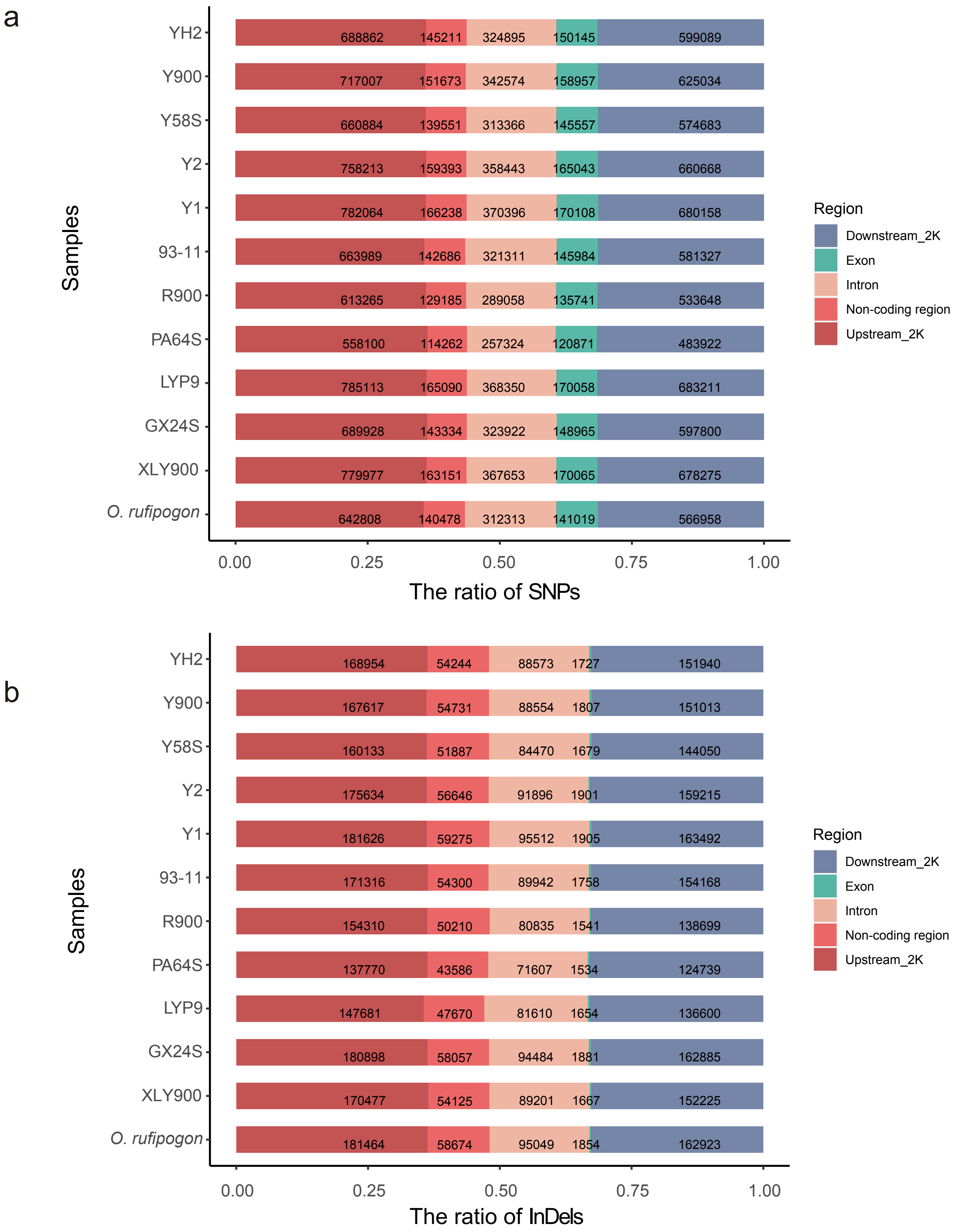

Figure S6. Distribution of genomic variations (SNPs and InDels) in different genomic regions of five super-hybrid rice varieties and their parental progenitors.

The figure illustrates the distribution of SNPs and InDels across various genomic regions; specifically, 2 kilobases (Kb) upstream and downstream of genes, exons, introns, and non-coding regions in five super-hybrid rice varieties alongside their respective hybrid parents. Panel (a) quantifies the ratio of SNPs, while panel (b) details the ratio of InDels, both of which are critical indicators of genetic diversity and evolution within these varieties. The data are represented as stacked bar graphs, with color coding to differentiate between the genomic regions.
