# Supplementary material for: The origin and evolution of cultivated rice and genomic signatures of heterosis for yield traits in super-hybrid rice
Source: BMC Biol. 2025 Jun 4;23:153. doi: 10.1186/s12915-025-02255-2 (PMC12139199; doi:10.1186/s12915-025-02255-2)
Supplement: Supplementary file 8 — Additional file 8: Fig. S7. Distribution of SVs in genomic regions of five super-hybrid rice varieties and their parental progenitors. Panel a and b of Fig. S7 depict the distribution of SVs, including deletions (DEL), duplications (DUP), insertions (INS), inversions (INV), and translocations (TRA), across different genomic regions of five super-hybrid rice varieties and their respective parental progenitors. These regions encompass two kilobases (kb) upstream and downstream of genes, as well as exons, introns, and intergenic regions. The data are represented in stacked bar charts for each sample, with colors indicating the type of SVs. [file 12915_2025_2255_MOESM8_ESM.pdf]

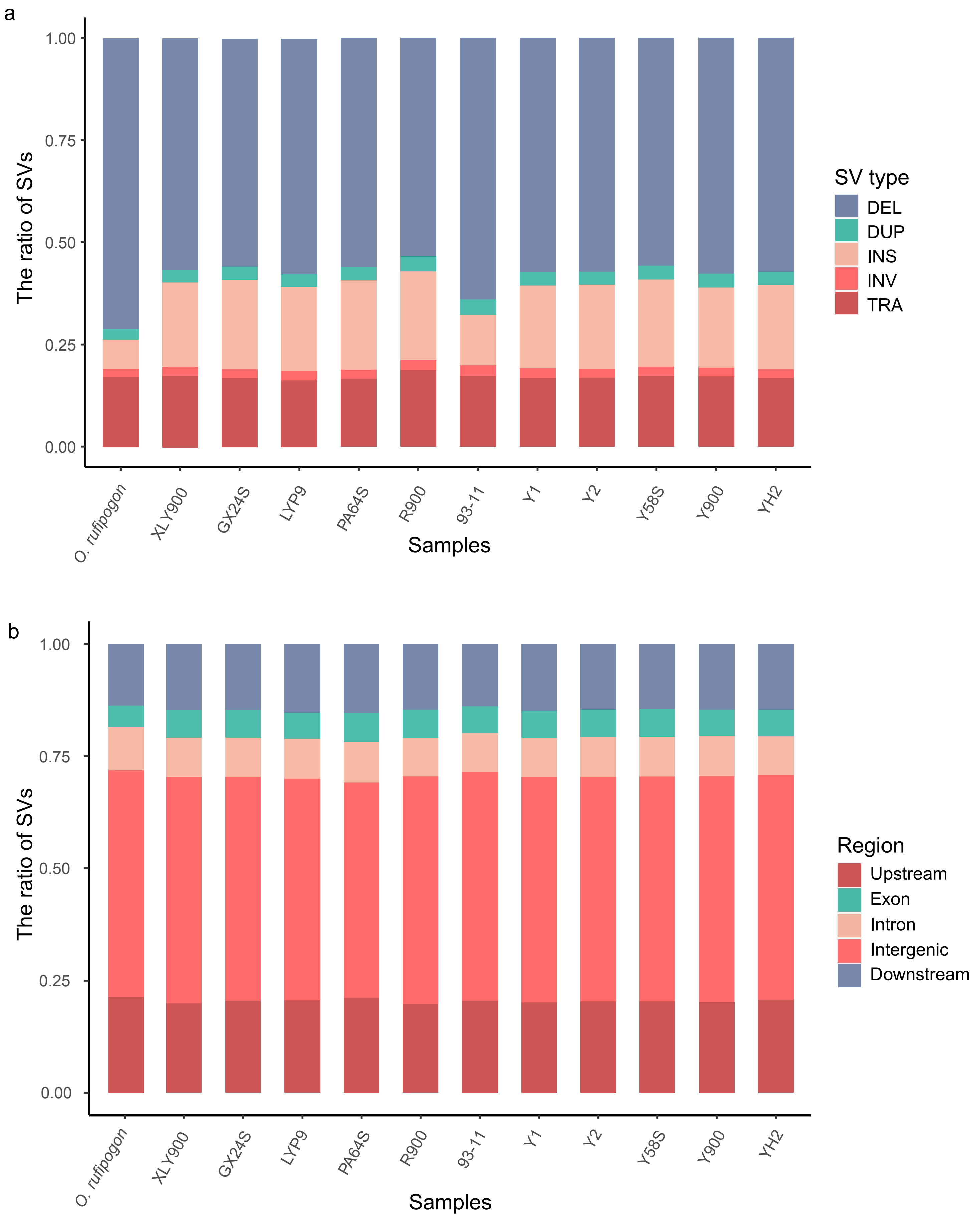

Figure S7. Distribution of SVs in genomic regions of five super-hybrid rice varieties and their parental progenitors.

Panel a and b of Figure S7 depict the distribution of structural variations (SVs), including deletions (DEL), duplications (DUP), insertions (INS), inversions (INV), and translocations (TRA), across different genomic regions of five super-hybrid rice varieties and their respective parental progenitors. These regions encompass two kilobases (Kb) upstream and downstream of genes, as well as exons, introns, and intergenic regions. The data are represented in stacked bar charts for each sample, with colors indicating the type of SVs.
