# Supplementary material for: The origin and evolution of cultivated rice and genomic signatures of heterosis for yield traits in super-hybrid rice
Source: BMC Biol. 2025 Jun 4;23:153. doi: 10.1186/s12915-025-02255-2 (PMC12139199; doi:10.1186/s12915-025-02255-2)
Supplement: Supplementary file 9 — Additional file 9: Fig. S8. Summary of the hybridization signal (γ) from HyDe analysis among the five super-hybrid rice varieties and their parental progenitors. Heatmap displays the hybridization signals detected by HyDe among five sets of super-hybrid rice and their parental progenitors, as well as the Nipponbare, with O. rufipogon as an outgroup. Each colored square on the heatmap corresponds to a hybridization signal identified by HyDe, with the intensity of color representing the genetic probability of the associated taxa on the Y-axis. [file 12915_2025_2255_MOESM9_ESM.pdf]

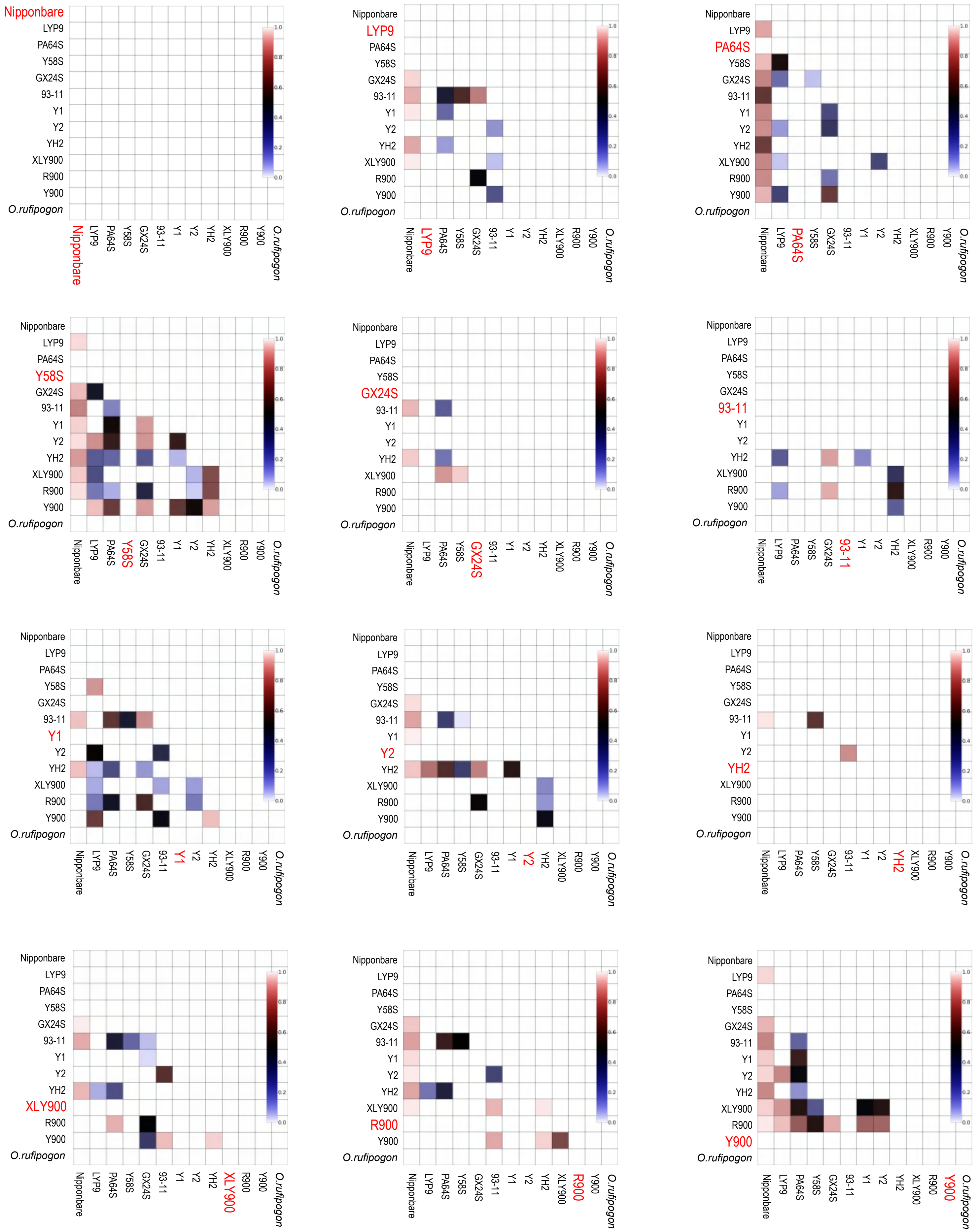


**Figure S8. Summary of the hybridization signal (γ) from HyDe analysis among the five super-hybrid rice varieties and their parental progenitors.**

Heatmap displays the hybridization signals detected by HyDe among five sets of super-hybrid rice and their parental progenitors, as well as the Nipponbare, with *O. rufipogon* as an outgroup. Each colored square on the heatmap corresponds to a hybridization signal identified by HyDe, with the intensity of color representing the genetic probability of the associated taxa on the Y-axis.
