# Supplementary material for: The origin and evolution of cultivated rice and genomic signatures of heterosis for yield traits in super-hybrid rice
Source: BMC Biol. 2025 Jun 4;23:153. doi: 10.1186/s12915-025-02255-2 (PMC12139199; doi:10.1186/s12915-025-02255-2)
Supplement: Supplementary file 10 — Additional file 10: Fig. S9. A Venn diagram summarizing overlapping eQTL loci among three super-rice varieties (LYP9, Y900, and XLY900) and their parental progenitors. [file 12915_2025_2255_MOESM10_ESM.pdf]

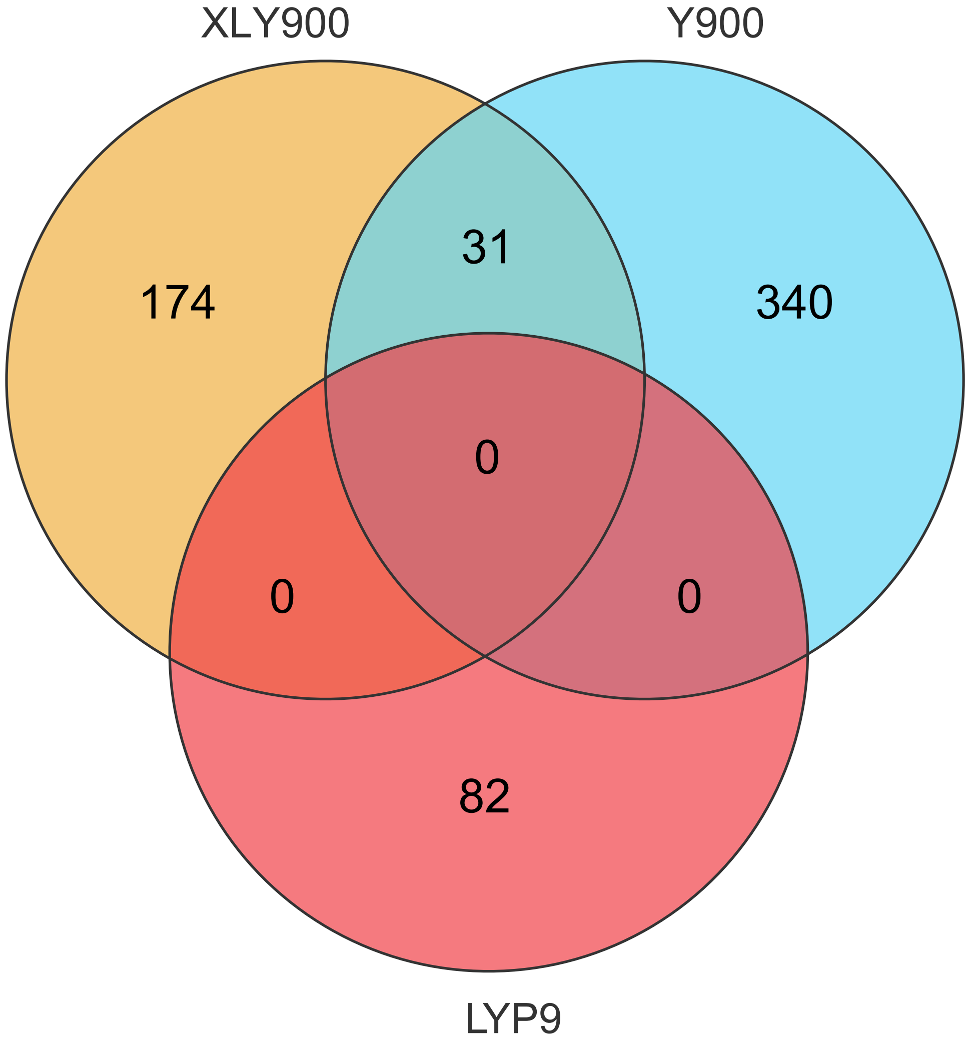

Figure S9. A Venn diagram summarizing overlapping eQTL loci among three super-rice varieties (LYP9, Y900, and XLY900) and their parental progenitors.
