# Supplementary material for: The origin and evolution of cultivated rice and genomic signatures of heterosis for yield traits in super-hybrid rice
Source: BMC Biol. 2025 Jun 4;23:153. doi: 10.1186/s12915-025-02255-2 (PMC12139199; doi:10.1186/s12915-025-02255-2)
Supplement: Supplementary file 11 — Additional file 11: Fig. S10. The bubble plot summarizing the biological processes of eQTL genes for three super-hybrid rice varieties and their parental progenitors based on Gene Ontology (GO). The figure summarizes the annotation of important biological processes (BP) terms (P-value < 0.05) for eQTL genes in the LYP9, Y900, and XLY900 super-hybrid rice varieties and their parental progenitors based on the Gene Ontology (GO) database. [file 12915_2025_2255_MOESM11_ESM.pdf]

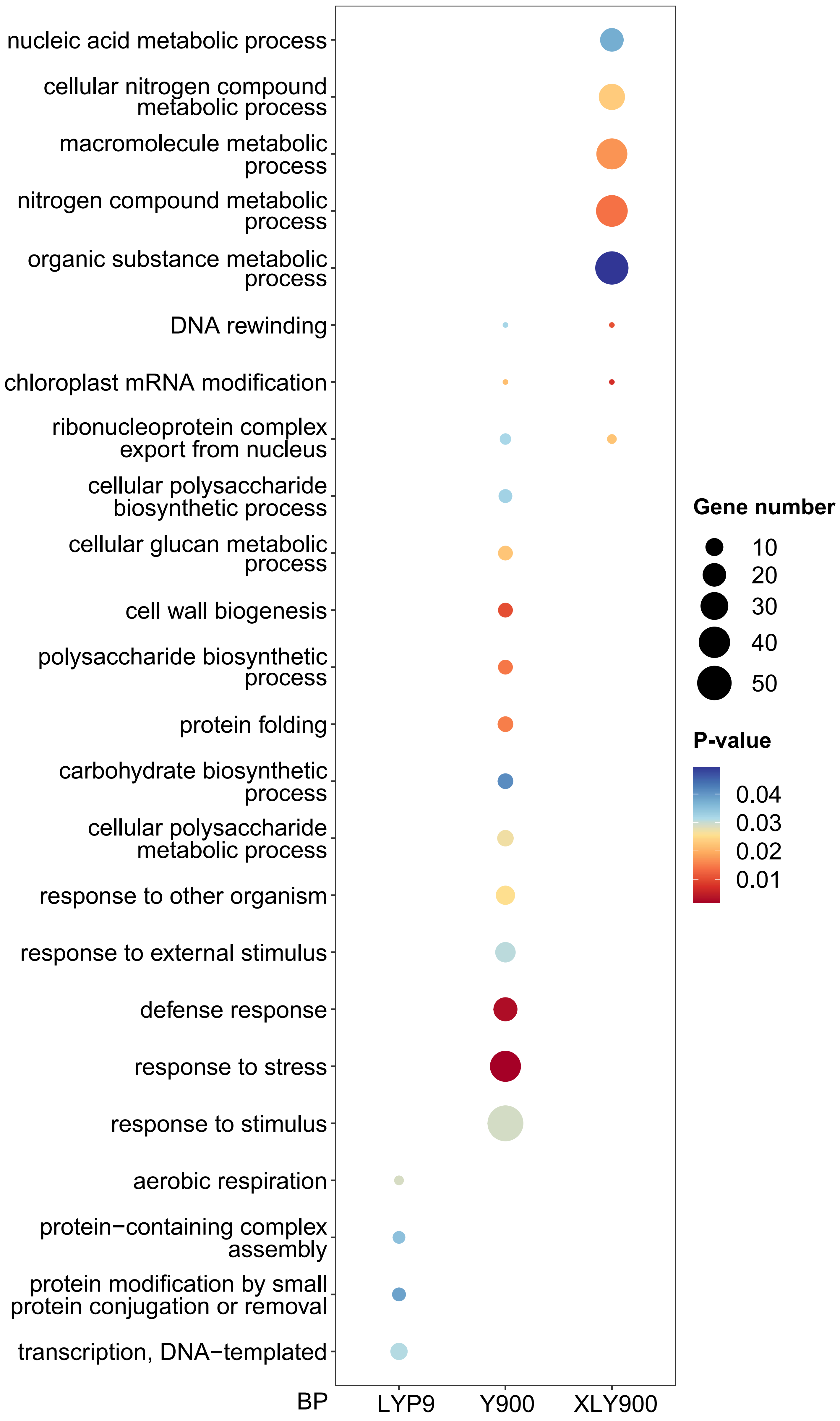

Figure S10. The bubble plot summarizing the biological processes of eQTL genes for three super-hybrid rice varieties and their parental progenitors based on Gene Ontology (GO).
The figure summarizes the annotation of important biological processes (BP) terms (P-value < 0.05) for eQTL genes in the LYP9, Y900, and XLY900 super-hybrid rice varieties and their parental progenitors based on the Gene Ontology (GO) database.
