# Supplementary material for: The origin and evolution of cultivated rice and genomic signatures of heterosis for yield traits in super-hybrid rice
Source: BMC Biol. 2025 Jun 4;23:153. doi: 10.1186/s12915-025-02255-2 (PMC12139199; doi:10.1186/s12915-025-02255-2)
Supplement: Supplementary file 12 — Additional file 12: Fig. S11. The bubble plot summarizes the KEGG pathway annotations (P-value < 0.05) for eQTL genes within the LYP9, Y900, and XLY900 super-hybrid rice varieties and their parental progenitors. [file 12915_2025_2255_MOESM12_ESM.pdf]

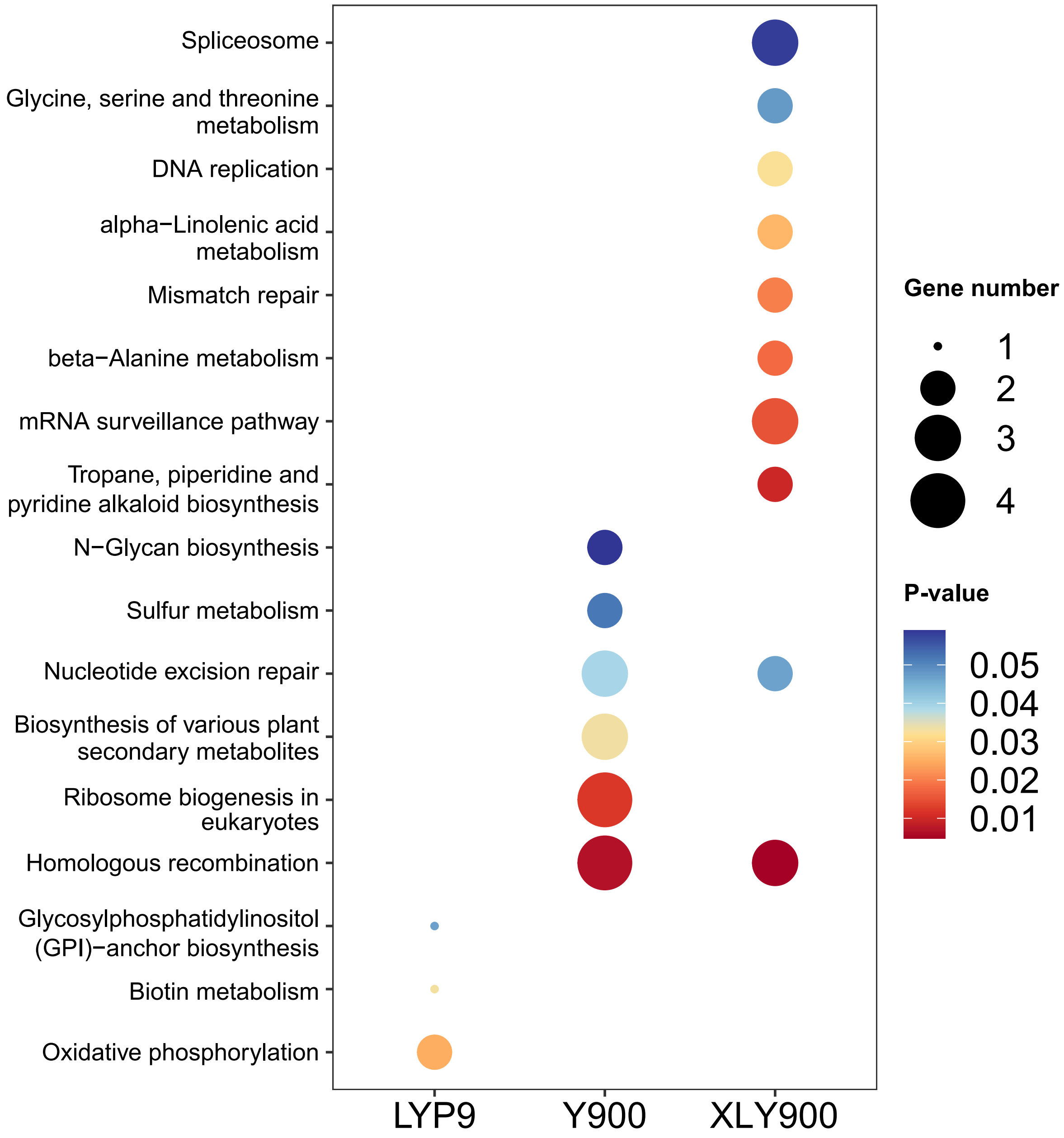

Figure S11. The bubble plot summarizes the KEGG pathway annotations (P-value < 0.05) for eQTL genes within the LYP9, Y900, and XLY900 super-hybrid rice varieties and their parental progenitors.
